# Palladium-based coordination cages as dynamic crosslinks in acrylamide hydrogels†

**DOI:** 10.1039/d5sc00335k

**Published:** 2025-02-27

**Authors:** Chaolei Hu, Damien W. Chen, Sylvain Sudan, Kay Severin

**Affiliations:** a Institut des Sciences et Ingénierie Chimiques, École Polytechnique Fédérale de Lausanne (EPFL) 1015 Lausanne Switzerland kay.severin@epfl.ch

## Abstract

Soft polymer networks with palladium cages as crosslinks can be obtained by combining polymeric N-donor ligands with Pd^II^ salts in organic solvents. Herein, we describe an alternative procedure that enables the preparation of hydrogels with Pd_*n*_L_2*n*_-type junctions. The gels were obtained by photoinitiated copolymerization of palladium cages with acrylamide monomers in water. Cages with varying nuclearities (*n* = 2, 4, or 12) and different acrylates were employed. The material properties could be tuned by changing the crosslinker density. Thermoresponsive hydrogels were obtained when NIPAm was used as the monomer. The dynamic nature of the Pd-based crosslinks allows the creation of stimuli-responsive hydrogels. In particular, we were able to alter the network topology of a hydrogel by anion-induced conversion of Pd_4_L_8_ crosslinks into Pd_2_L_4_-type junctions.

## Abstract 2

Hydrogels with Pd_*n*_L_2*n*_-type junctions can be obtained by photoinitiated copolymerization of palladium cages with acrylamide monomers in water. The dynamic nature of the cage crosslinks allows for an anion-induced switch in network topology.

## Graphical abstract

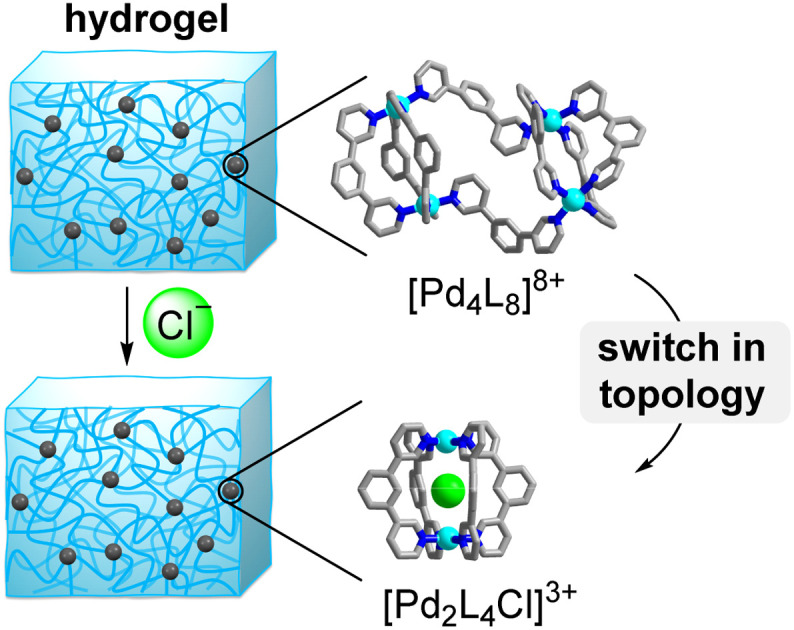

## Introduction

Palladium-based coordination cages of the general formula [Pd_*n*_L_2*n*_]X_2*n*_ (X = weakly coordinating anion) can be obtained by thermal equilibration of Pd^II^ salts with ditopic nitrogen-donor ligands.^1^ The Pd–N bonds in these assemblies are strong enough to ensure that the cages are stable in coordinating solvents. Despite their high stability, Pd–N bonds remain sufficiently labile, allowing for stimuli-induced structural rearrangements. Changes in the nuclearity *n* of Pd_*n*_L_2*n*_ complexes have been achieved with light,^2^ by a change of the solvent,^3^ or by the addition of chemical templates.^4^

Another interesting feature of Pd_*n*_L_2*n*_-based coordination cages is their ability to serve as hosts for different guests. The versatile host–guest chemistry has enabled applications in fields such as catalysis^5^ or medicinal chemistry.^6^

**Fig. 1:**
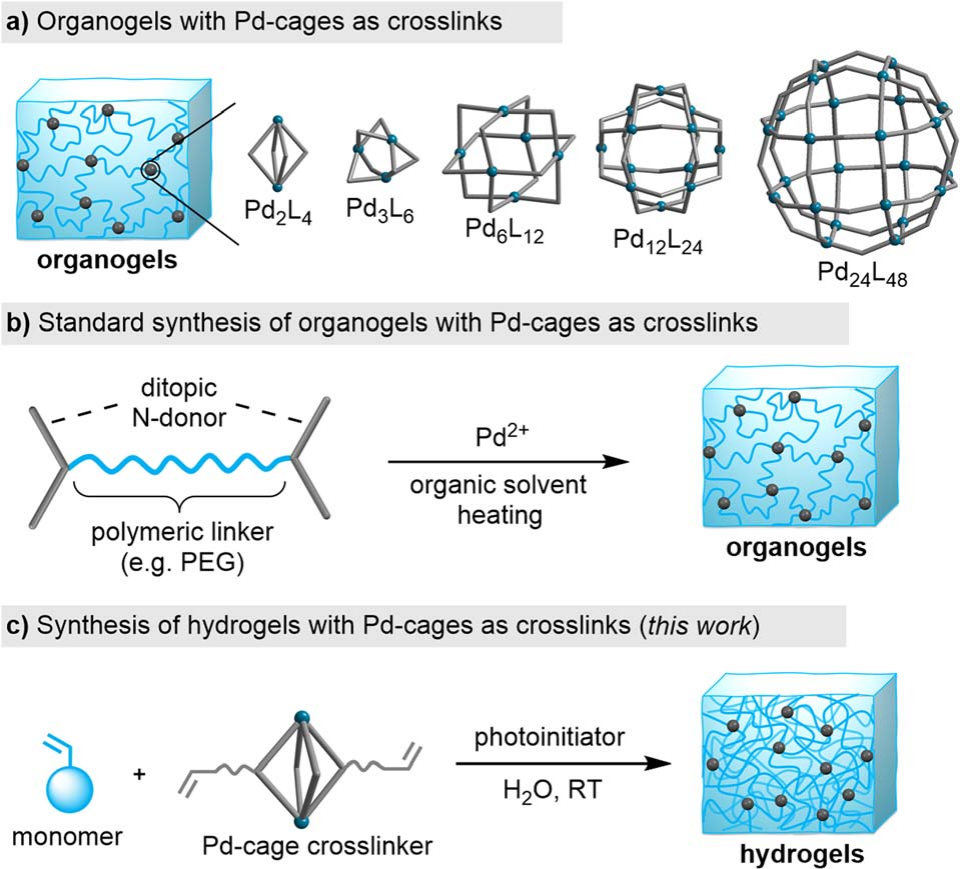
(a) Pd_*n*_L_2*n*_-type cages with nuclearities ranging from *n* = 2 to 24 have been used as crosslinks in organogels. (b) The standard synthesis of organogels with Pd-cages as crosslinks. (c) Synthesis of hydrogels by copolymerization of monomers with Pd-cages featuring acrylate side chains (this work).

Traditionally, coordination cages (also referred to as ‘metal–organic cages’) have been studied primarily in solution and in the crystalline state. However, these metallosupramolecular structures are increasingly being explored within the context of materials science, with a special focus on polymer networks.^7^ Pd_*n*_L_2*n*_-type cages with nuclearities ranging from *n* = 2 to 24 have been incorporated into organogels (Fig. 1a).^7–11^ The typical synthetic route for producing such gels involves using N-donor ligands, which are connected by a polymeric linker (*e.g.* a polyethylene glycol chain). The polymer networks are then obtained by combining the ligand with a Pd^II^ salt in an organic solvent at slightly elevated temperatures (Fig. 1b).

Organogels with Pd_*n*_L_2*n*_-type cages as crosslinks can display high branch functionalities^8^ and the material properties are broadly tunable.^9^ The dynamic nature of the Pd–N bonds enables the formation of stimuli-responsive gels. For example, light-responsive gels were obtained by using photochromic N-donor ligands^10^ or external photoacids.^11^ Moreover, the gel properties can be altered by adding suited guest molecules.^12^

We sought to investigate whether Pd_*n*_L_2*n*_ cages could be incorporated into other types of gel matrices, particularly acrylamide hydrogels.^13^ The thermal stability of Pd^II^ salts in water poses a challenge, as heating can lead to their decomposition. This makes the thermal equilibration of polymeric ligands with Pd^II^ salts potentially problematic. Indeed, our initial attempts to synthesize hydrogels using polyethylene glycol-linked dipyridyl ligands were compromised by the apparent formation of metallic Pd during the heating of the ligand/Pd^II^ mixture in water, as indicated by the coloration of the solution (see the ESI, Fig. S17†).

In view of these difficulties, we decide to explore an alternative approach: the copolymerization of acrylamide monomers with pre-formed Pd_*n*_L_2*n*_ cages having polymerizable side chains. We have recently shown that this strategy can be employed for the synthesis of nanogels.^14^ We now demonstrate that bulk acrylamide hydrogels can be obtained by photoinitiated copolymerization of di-, tetra-, and dodecanuclear Pd cages with different acrylamide monomers (Fig. 1c). A post-synthetic change in the network topology was achieved by guest-induced rearrangement of Pd_4_L_8_ crosslinks into Pd_2_L_4_ connections.

## Results and discussion

**Scheme 1:**
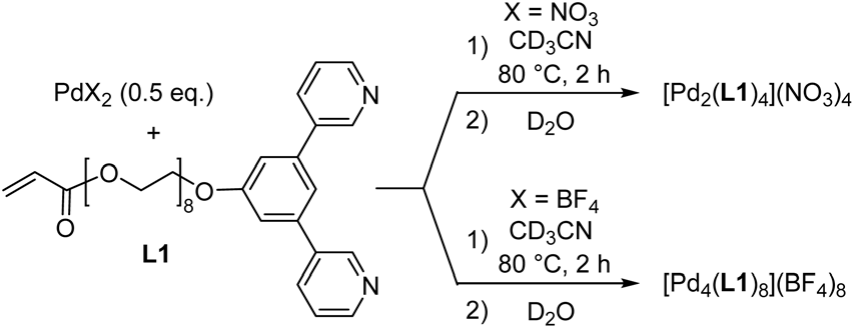
Synthesis of the complexes [Pd_2_(L1)_4_](NO_3_)_4_ and [Pd_4_(L1)_8_](BF_4_)_8_.

For the synthesis of hydrogels with Pd_*n*_L_2*n*_-based coordination cages, we needed water-soluble cages with polymerizable side chains. Toward this goal, we first prepared the dipyridyl ligand L1 (Scheme 1). The synthesis of L1 was accomplished in four steps with an overall yield of 51% (for details, see the ESI, Section 2.1†). The PEG_8_ chain of L1 was expected to provide solubility in water, while the terminal acrylate group could be used for copolymerization reactions.

Ligand L1 was then combined with 0.5 equivalents of Pd(NO_3_)_2_ in CD_3_CN. After heating the mixture for two hours at 80 °C, the ^1^H NMR spectrum indicated the formation of a highly symmetric complex (Fig. S18†). Analysis of the product by mass spectrometry confirmed that the expected dinuclear cage [Pd_2_(L1)_4_](NO_3_)_4_ had formed (Fig. S20†).

Following synthesis and analysis in CD_3_CN, the solvent was exchanged for D_2_O. Complex [Pd_2_(L1)_4_](NO_3_)_4_ could be re-dissolved without problems at 5 mM concentrations, and the analytical data confirmed that the assembly had not undergone any structural changes upon switching the solvent (Fig. S24†).

**Fig. 2:**
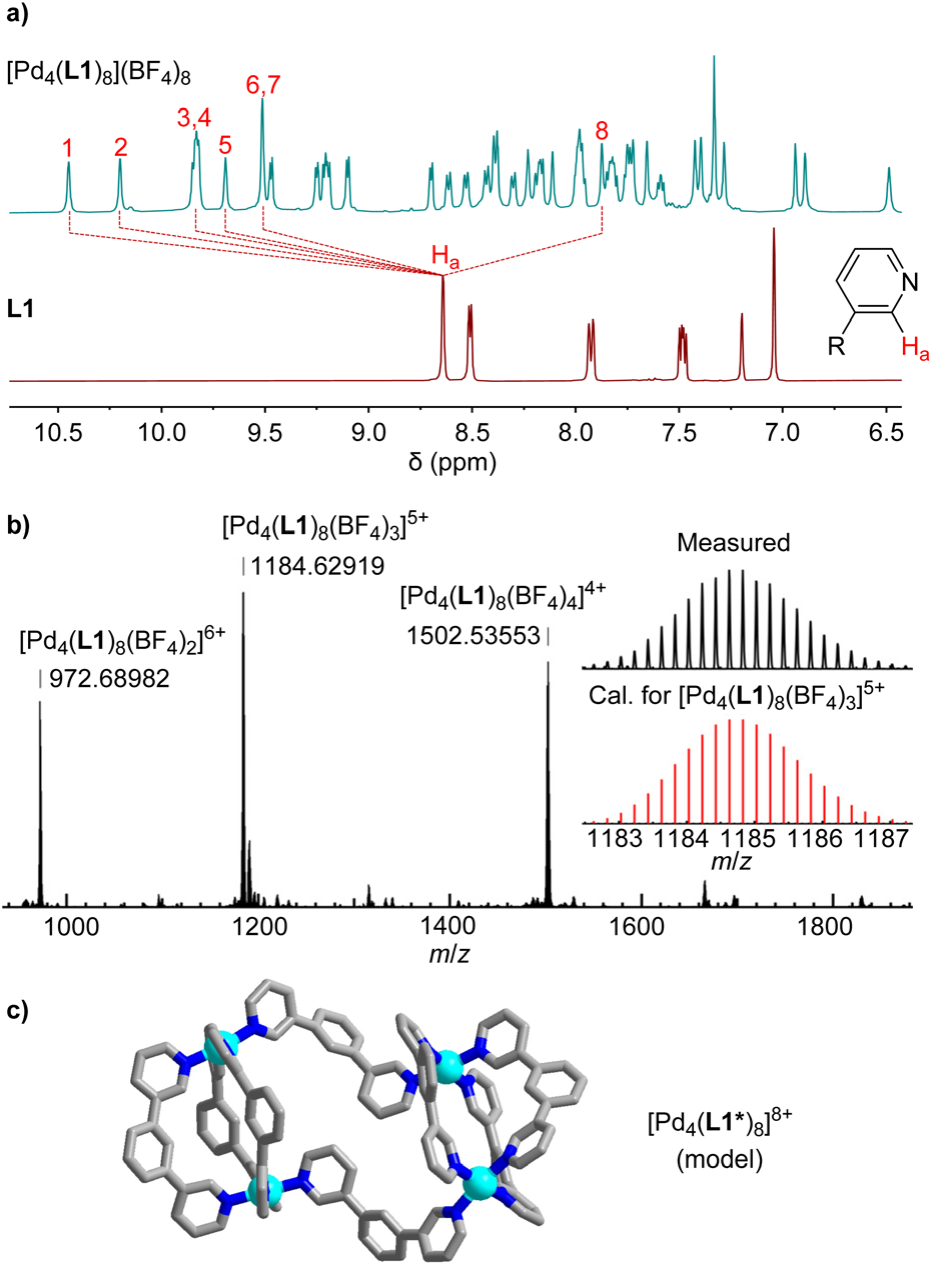
(a) ^1^H NMR spectra (400 MHz, D_2_O) of [Pd_4_(L1)_8_](BF_4_)_8_ and of ligand L1. (b) High resolution ESI mass spectrum of [Pd_4_(L1)_8_](BF_4_)_8_. (c) Molecular model of [Pd_4_(L1*)_8_]^8+^ (the side chains were omitted).

When ligand L1 was combined with [Pd(CH_3_CN)_4_](BF_4_)_2_ instead of Pd(NO_3_)_2_, a different outcome was observed. The ^1^H NMR spectrum of the CD_3_CN solution after thermal equilibration was very complex. Moreover, the spectrum was found to be concentration- and temperature-dependent. After solvent switch to D_2_O, the spectrum revealed the presence of a defined, low-symmetry assembly (Fig. 2a). The mass spectrum showed that this species was a tetranuclear complex (Fig. 2b).

The two most common structural motifs for Pd_4_L_8_ complexes are macrocyclic^15^ and tetrahedral.^16^ Furthermore, there are examples of interlocked (Pd_2_L_4_)_2_ cages.^17^ Recently, we described a fourth structural motif: a doubly-bridged (Pd_2_L_3_)(μ-L)_2_(Pd_2_L_3_) cage, the formation of which was templated by an ion pair.^18^ The low apparent symmetry of [Pd_4_(L1)_8_](BF_4_)_8_, with an eightfold splitting of the ligands signals (Fig. 2a), suggested that [Pd_4_(L1)_8_](BF_4_)_8_ displays a (Pd_2_L_3_)(μ-L)_2_(Pd_2_L_3_)-type of structure. Molecular modeling of [Pd_4_(L1)_8_](BF_4_)_8_, excluding the PEG side chains, revealed that the ligands adopt two distinct conformations. A convergent orientation of the pyridyl groups is observed for the ligands within the Pd_2_(L1)_3_ units, and a divergent orientation of the N-donor groups is found for the ligands bridging the two Pd_2_(L1)_3_ units (Fig. 2c).

The striking difference between the reactions with Pd(NO_3_)_2_ and [Pd(CH_3_CN)_4_](BF_4_)_2_ can be attributed to the fact that nitrate is able to template the formation of Pd_2_L_4_ cages with bridging 1,3-di(pyridin-3-yl)benzene ligands (one nitrate anion is encapsulated in the cage cavity).^19^ The BF_4_ anion is less suited as a template, and the inherent strain of the dinuclear Pd_2_(L1)_4_ complex^19*b*^ cannot be compensated. As a result, the system rearranges to give an entropically disfavored but less strained tetranuclear assembly.

**Scheme 2:**
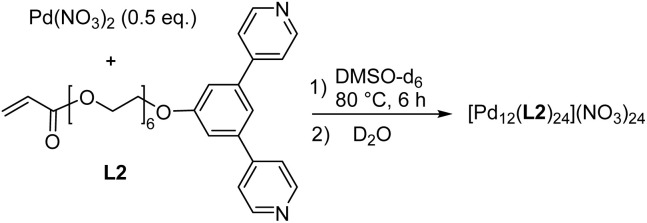
Synthesis of the complexes [Pd_12_(L2)_24_](NO_3_)_24_.

In addition to the di- and tetranuclear complexes [Pd_2_(L1)_4_](NO_3_)_4_ and [Pd_4_(L1)_8_](BF_4_)_8_, we synthesized the dodecanuclear cage [Pd_12_(L2)_24_](NO_3_)_24_ (Scheme 2). The required ligand L2 was obtained in four steps following a related synthetic procedure as used for L1 (for details, see the ESI, Section 2.1†). The 1,3-di(pyridin-4-yl)benzene part in ligand L2 is well known to promote the formation of cuboctahedral Pd^II^ cages,^20^ and the analytical data of [Pd_12_(L2)_24_](NO_3_)_24_ are in line with the anticipated structure (for details, see the ESI, Section 2.5†).

**Fig. 3:**
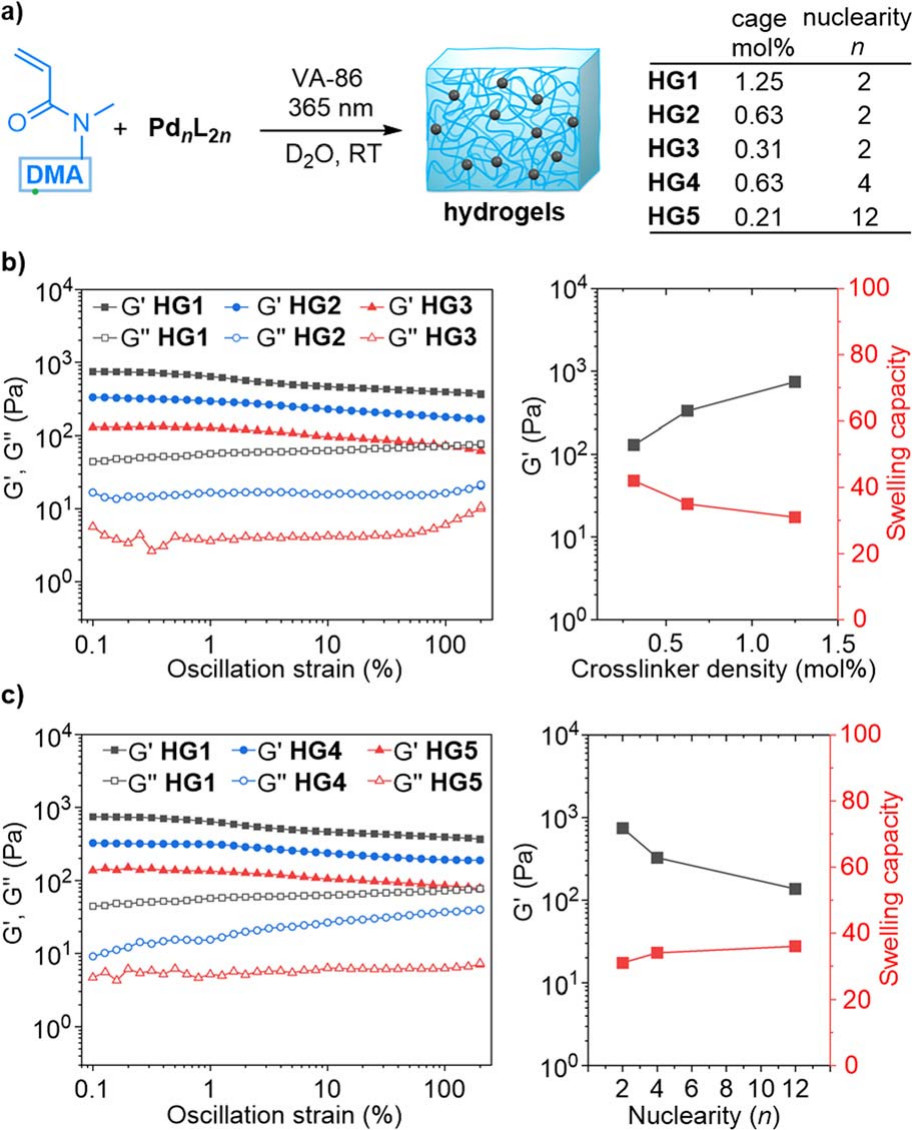
(a) Synthesis of the hydrogels HG1–HG5 by photoinitiated copolymerization of DMA with [Pd_2_(L1)_4_](NO_3_)_4_, [Pd_4_(L1)_8_](BF_4_)_8_, or [Pd_12_(L2)_24_](BF_4_)_24_. (b) Rheological data and swelling capacities of gels with different crosslinker densities (HG1–HG3). (c) Rheological data and swelling capacities of gels with different cage crosslinks (HG1, HG4, and HG5).

Next, we examined if the three cages could be employed as crosslinks for the synthesis of hydrogels. Initial experiments were carried out with the dinuclear cage [Pd_2_(L1)_4_](NO_3_)_4_ and *N*,*N*-dimethylacrylamide (DMA) as the monomer. Mixtures of DMA and the cage were copolymerized in D_2_O by irradiation at 365 nm using 2,2′-azobis-[2-methyl-*N*-(2-hydroxyethyl)-propionamide] (VA-86) as photoinitiator. Three different cage concentrations were employed: 0.31, 0.63, or 1.25 mol% with respect to the monomer concentration (400 mM). All three reactions resulted in the formation of hydrogels (HG1–HG3, Fig. 3 and S36†).

The ^1^H NMR spectra of the gels showed broad peaks in the aromatic region, which matched the signals observed for [Pd_2_(L1)_4_](NO_3_)_4_ (Fig. S37–39†). The data suggest that the polymerization had not compromised the cage structures.

The mechanical properties of the gels HG1–HG3 were evaluated by oscillatory rheometry. Amplitude sweep tests at 1 rad s^−1^ were performed with the swollen hydrogels. Over the entire range of oscillation strain, the storage moduli (*G*′) were higher than the loss moduli (*G*′′), indicating a strong elastic response from the gels (Fig. 3b). The stiffness of the hydrogel was found to depend on the crosslinker density. The *G*′ (at 0.1% strain) of the hydrogel increased from a value of 130 Pa for HG3 (0.31 mol% cage) to a value of 743 Pa for HG1 (1.25 mol% cage) (Fig. 3b). Moreover, a higher cage concentration was found to reduce the swelling capacity of the hydrogels (Fig. 3b).

Subsequently, we investigated the influence of the cage nuclearity *n* on the gel properties. The tetranuclear cage [Pd_4_(L1)_8_](BF_4_)_8_ and the dodecanuclear cage [Pd_12_(L2)_24_](NO_3_)_24_ were copolymerized with DMA to give the hydrogels HG4 and HG5, respectively (Fig. 3a). The cage concentrations were chosen so that the gels HG1, HG4, and HG5 all contain the same amount of Pd and ligand L1/L2. As a result, the branch functionality of the gels was expected to increase in the order HG1 < HG4 < HG5, while the concentration of the cages was expected to decrease in the same order.

Rheology measurements revealed an indirect correlation between the nuclearity *n* of the cage crosslinker and the storage modulus: the *G*′ values of the hydrogels dropped from 743 Pa for HG1 (*n* = 2), to 326 Pa for HG4 (*n* = 4), and to 136 Pa for HG5 (*n* = 12) (Fig. 3c). The reduced branch concentrations for gels based on cages with a higher nuclearity *n* are likely responsible for the reduced gel stiffness. As before, we found that the softer gels displayed a higher swelling capacity (Fig. 3c).

In addition to DMA, we have used *N*-hydroxyethyl acrylamide (HEAA) and *N*-isopropylacrylamide (NIPAm) as monomers in copolymerization reactions with the dinuclear complex [Pd_2_(L1)_4_](NO_3_)_4_. In order to allow a comparison with HG1, we have employed a uniform cage concentration of 1.25 mol%.

**Fig. 4:**
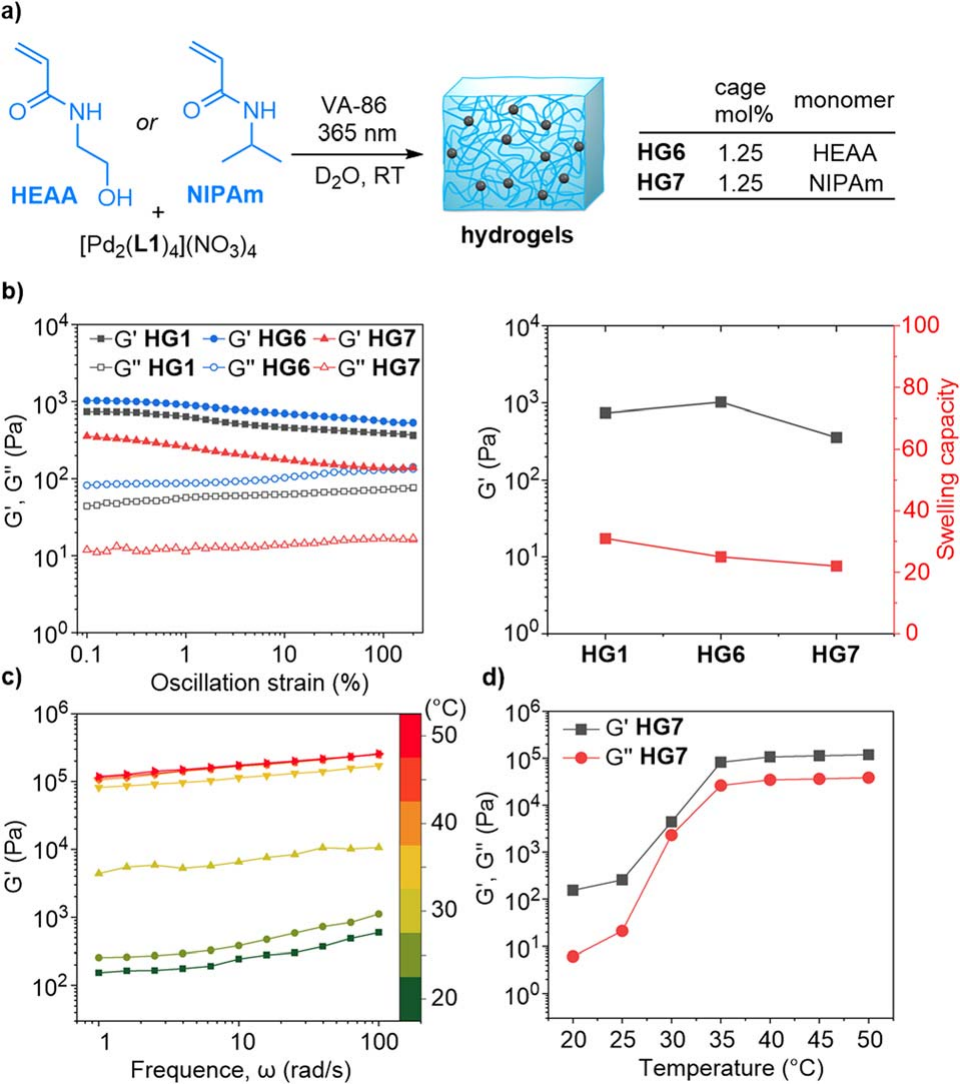
(a) Synthesis of the hydrogels HG6 and HG7 by photoinitiated copolymerization of HEAA or NIPAm with [Pd_2_(L1)_4_](NO_3_)_4_. (b) Rheological data and swelling capacities of gels based on different monomers (HG1, HG6, and HG7). (c) Storage moduli *G*′ of HG7 as a function of the angular frequency at different temperatures. (d) Storage moduli *G*′ and loss moduli *G*′′ (at 1 rad s^−1^) of HG7 at different temperatures.

The polymerizations with HEAA and NIPAm gave stable gels (HG6 and HG7, Fig. 4a and S47†). The highest storage modulus at 0.1% strain was observed for HG6 (1022 Pa), followed by HG1 (743 Pa), and then HG7 (358 Pa). The swelling capacity of HG6 and HG7 was found to be lower than that of HG1 (Fig. 4b).

Polymers based on NIPAm tend to be thermoresponsive.^21^ To examine if HG7 would display a temperature-dependent behavior, we performed frequency sweep tests at 1% strain at different temperatures. The storage and loss moduli were found to increase with temperature (Fig. 4c and d). The lower critical solution temperature (LCST) of ∼30 °C is in the typical range for NIPAm-based hydrogels.^21^

**Fig. 5:**
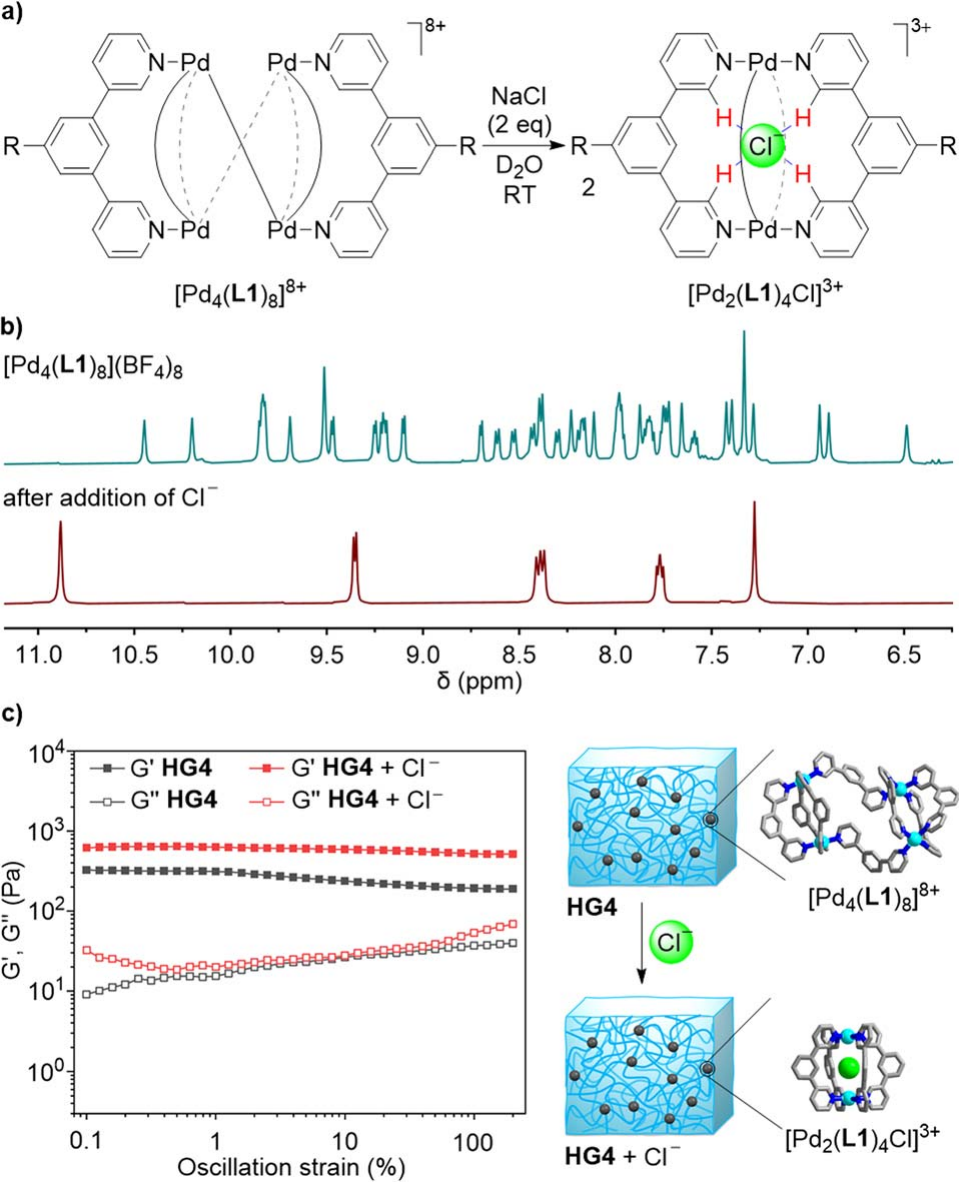
(a) Chloride-induced rearrangement of [Pd_4_(L1)_8_]^8+^ into [Pd_2_(L1)_4_Cl]^3+^. (b) ^1^H NMR spectra (400 MHz, D_2_O) of a solution of [Pd_4_(L1)_8_](BF_4_)_8_ in D_2_O before (top) and after chloride-induced rearrangement (bottom). (c) Rheological data of HG4 before and after treatment with chloride.

As discussed above, Pd_2_L_4_ cages with bridging 1,3-di(pyridin-3-yl)benzene ligands can bind nitrate. Cages of this type display an even higher affinity for chloride.^19*a*^ Since the tetranuclear complex [Pd_4_(L1)_8_](BF_4_)_8_ contains bridging 1,3-di(pyridin-3-yl)benzene ligands, we hypothesized that chloride could induce a rearrangement into a dinuclear complex. Indeed, when two equivalents of NaCl were added to a solution of [Pd_4_(L1)_8_](BF_4_)_8_ in D_2_O (2.5 mM), a quantitative rearrangement into a highly symmetric complex was observed by ^1^H NMR spectroscopy (Fig. 5b). An analysis by mass spectrometry confirmed that the chloride adduct [Pd_2_(L1)_4_Cl]^3+^ had formed (Fig. 5a).

The possibility to cleanly convert the tetranuclear complex [Pd_4_(L1)_8_](BF_4_)_8_ into the dinuclear complex [Pd_2_(L1)_4_Cl]^3+^ in homogenous solution suggested that a similar rearrangement could be achieved in the gel state. Two equivalents of NaCl (with respect to cage crosslinks) were added to HG4 and the mixture was incubated for two days at room temperature. Analysis of the gel by ^1^H NMR spectroscopy confirmed that a rearrangement had occurred (see the ESI, Fig. S54 and S55†). The mechanical properties of the new gel were examined by oscillatory rheometry. The result showed that the chloride-induced rearrangement had increased the stiffness of the gel (Fig. 5c). This finding can be explained by the fact that the concentration of the crosslinks in the gels has increased. The mechanical properties of HG4 after incubation with chloride were similar to what was observed for HG1: the storage modulus at 0.1% strain for HG4 + Cl^−^ was 650 Pa, whereas a value of 743 Pa was found for HG1.

## Conclusions

Thus far, soft polymer networks with palladium-based cages as crosslinks were primarily obtained by combining polymeric N-donor ligands with palladium(ii) salts in organic solvents. We have introduced an alternative method for creating gels with Pd_*n*_L_2*n*_-type junctions. This approach involves the photo-induced copolymerization of palladium cages with acrylamide monomers in water, providing an efficient pathway to synthesize bulk hydrogels. The method is versatile, allowing for the use of palladium cages with varying nuclearities (*n* = 2, 4, or 12), as well as different types of acrylamides.

By controlling the density of the palladium cages in the network, we were able to change the material properties of the resulting hydrogels. When *N*-isopropylacrylamide was used as the monomer, a thermoresponsive hydrogel was obtained. The dynamic nature of the Pd-based crosslinks enables the development of stimuli-responsive hydrogels. Specifically, we showed that the material properties of a hydrogel could be altered by the addition of chloride. The observed changes are likely due to a conversion of Pd_4_L_8_ crosslinks into Pd_2_L_4_-type junctions. The possibility of modifying the gel properties *via* dynamic metallosupramolecular chemistry represents an interesting feature of hydrogels based on Pd cages.

## Data availability

The data supporting this article have been included as part of the ESI.†

## Author contributions

C. H. and K. S. initiated the study, C. H. performed the experiments and analyzed the data, D. W. C. carried out the molecular modelling, S. S. performed initial studies with a Pd_4_L_8_ complex, and C. H. and K. S. co-wrote the manuscript. All authors discussed the results and commented on the manuscript.

## Conflicts of interest

There are no conflicts to declare.

## Supplementary Material

SC-OLF-D5SC00335K-s001
