# Supplementary material for: Palladium-based coordination cages as dynamic crosslinks in acrylamide hydrogels
Source: Chem Sci. 2025 Feb 27;16(13):5559–64. doi: 10.1039/d5sc00335k (PMC11865919; doi:10.1039/d5sc00335k)
Supplement: SC-016-D5SC00335K-s001 [file SC-016-D5SC00335K-s001.pdf]

Supplementary Information (SI) for Chemical Science.
This journal is © The Royal Society of Chemistry 2025

Electronic Supplementary Information

Palladium-based coordination cages as dynamic crosslinks in acrylamide hydrogels

Chaolei Hu, Damien W. Chen, Sylvain Sudan, and Kay Severin*

Institut des Sciences et Ingénierie Chimiques, École Polytechnique Fédérale de Lausanne (EPFL), 1015 Lausanne, Switzerland

Table of Contents

Table of Contents 2
1 Materials and methods 3
1.1 Materials 3
1.2 Methods 3
2 Synthesis and characterizations 4
2.1 Synthesis and characterization of ligand L1 and L2 4
Synthesis of compounds 3 and 4 4
Synthesis of compounds 6 and 7 6
Synthesis of compound 10 8
Synthesis of compound 11 9
Synthesis of compounds L1 and L2 10
2.2 Synthesis of hydrogel HG0 by Pd^{2+}-mediated crosslinking of a polymeric ligand 12
2.3 Synthesis and characterization of cage $[Pd_2(L1)_4](NO_3)_4$ 13
2.4 Synthesis and characterization of cage $[Pd_4(L1)_8]$ $(BF_4)_8$ 16
2.5 Synthesis and characterization of cage $[Pd_{12}(L2)_{24}](NO_3)_{24}$ 19
2.6 Synthesis and characterization of the hydrogels HG1–HG3 22
2.7 Synthesis and characterization of hydrogels HG4 and HG5 25
2.8 Synthesis and characterization of the hydrogels HG6 and HG7 27
3 Chloride-triggered rearrangement of $[Pd_4(L1)_8]^{8+}$ into $[Pd_2(L1)_4Cl]^{3+}$ 29
4 Chloride-triggered topology changes of HG4 30
5 Modeling details 33
6 References 37

1 Materials and methods

1.1 Materials

Octaethylene glycol (IVALUA, 99%), hexaethylene glycol (Sigma-Aldrich, 99%), *p*-toluolsulfonylchlorid (Sigma-Aldrich, 99%), KI (Sigma-Aldrich, 99%), Ag_2O (Sigma-Aldrich, 99%), 3,5-dibromophenol (Sigma-Aldrich, 97%), $Cs_2(CO_3)$ (Sigma-Aldrich, 99%), 3-pyridineboronic acid pinacol ester (Sigma-Aldrich, 97%), 4-pyridineboronic acid pinacol ester (Sigma-Aldrich, 97%), $Pd(PPh_3)_4$ (Sigma-Aldrich, 99%), K_2CO_3 (Sigma-Aldrich, 99%), acryloyl chloride (Sigma-Aldrich, 97%), triethylamine (Sigma-Aldrich, 99%), $Pd(NO_3)_2 \times 2\ H_2O$ (abcr, 99.9%, 41%Pd), $[Pd(CH_3CN)_4](BF_4)_2$ (abcr, 98%), *N*,*N*-dimethylacrylamide (DMA, Sigma-Aldrich, 99%), *N*-hydroxyethyl acrylamide (HEAA, Sigma-Aldrich, 97%), and photo-initiator VA-86 (Fujifilm, 98%) were used as received. *N*-Isopropylacrylamid (NIPAM, Sigma-Aldrich, 97%) was distilled and recrystallized from hexane before use. The polymeric ligand **L1’-PEG$_{4600}$-L1’** was synthesized following literature procedures.[1]

1.2 Methods

NMR spectra were measured on a Bruker Avance Neo spectrometer (^1H: 500 MHz, $^{13}C\{^1H\}$: 125 MHz) equipped with a $CPTCl_{xyz}$ 5 mm cryoprobe, a Bruker Avance III spectrometer (^1H: 400 MHz) equipped with a $BBFO_z$ 5 mm probe and a Bruker Avance III spectrometer (^1H: 400 MHz) equipped with a Prodigy BBO 5 mm cryoprobe. The chemical shifts are reported in part per million (ppm) using the solvent residual signal as a reference.

High-resolution mass spectrometry experiments were carried out using a hybrid ion trap-Orbitrap Fourier transform mass spectrometer, Orbitrap Elite (Thermo Scientific) equipped with a TriVersa Nanomate (Advion) nano-electrospray ionization source. Mass spectra were acquired with a minimum resolution setting of 120,000 at 400 *m*/*z*. To reduce the degree of analyte gas phase reactions leading to side products unrelated to solution phase, the transfer capillary temperature was lowered to 50 °C. Experimental parameters were controlled via standard and advanced data acquisition software.

UV irradiation for the photoinitiated polymerization was performed using a UV lamp (CAMAG UV Lamp, 365 nm, 40 W), and the irradiation distance was kept at 1 cm for all reactions.

Rheology was performed on a DHR-3 TA Instrument with an 8 mm diameter parallel plate steel geometry. The amplitude sweep measurements were performed from 0.1% to 200% strain at a constant frequency of 1 rad s^{-1}. Frequency sweeps were performed from 1 to 100 rad s^{-1} at 1% strain. For all measurements, the loaded hydrogel samples were immersed in mineral oil to reduce solvent evaporation and hydrogel de-swelling by adsorbing moisture, and the gap was kept constant at 800 µm.

The swelling ability of synthesized hydrogels were measured as follows. To each gel was added 1 mL water, and the gels were allowed to equilibrate at RT for 3 d, at which time the excess water was removed via syringe, and any residual water was wicked away by gently dabbing the gels in the vials. The hydrogels in the vials were weighed, and the dry mass of the gels were weighted after lyophilization. The swelling ratio for each hydrogel was determined by diving the mass of the swollen hydrogel by the dry mass of the hydrogel.

SEM measurements were carried out with freeze-dried hydrogel samples using a scanning electron microscope (Zeiss Merlin). The samples were coated with about 6 nm of iridium to make them conductive before measurements. The SEM electron beam accelerating voltage used was 30 kV.

2 Synthesis and characterizations

2.1 Synthesis and characterization of ligand L1 and L2

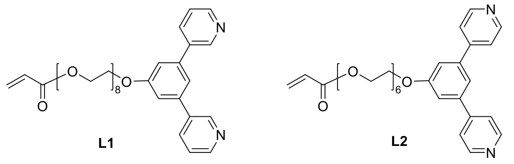

Scheme S1. Chemical structures of ligands **L1** and **L2**.

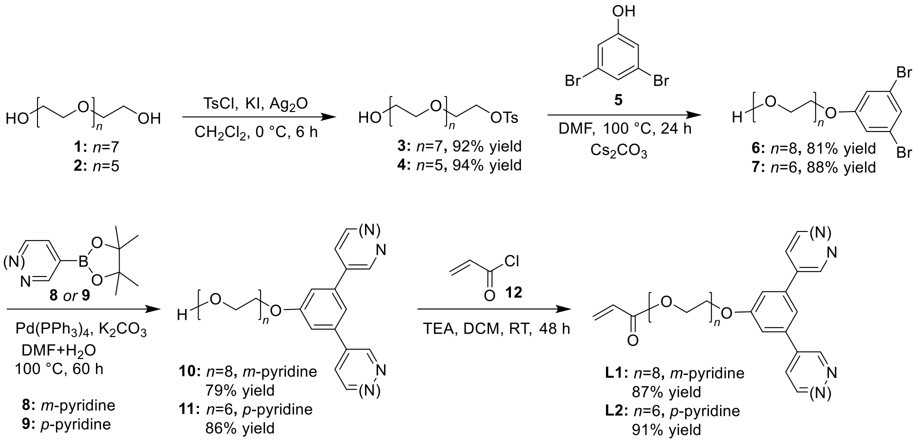

Scheme S2. Synthesis of the ligands **L1** and **L2**.

Synthesis of compounds 3 and 4

The synthesis of compounds **3** and **4** was adapted from a reported procedure.[2] Octaethylene glycol (**1**) or hexaethylene glycol (**2**) (1.0 equiv, 20 mmol) was dissolved in dry dichloromethane (150 mL), and the solution was cooled to 0 °C. Under vigorous stirring, Ag_2O (1.5 equiv, 30 mmol), *p*-toluolsulfonylchlorid (1.05 equiv, 21.0 mmol), and KI (0.2 equiv, 0.40 mmol) were added under inert conditions. After stirring for 2 h, the precipitated silver salts were removed by filtration through a pad of Celite, which was washed thoroughly with ethyl acetate. The combined filtrate was concentrated under vacuum, and the residue was purified by column chromatography (**3**: hexane: ethyl acetate, 1:20; **4**: hexane: ethyl acetate, 1:20). Compound **3** was obtained as a colorless oil (9.70 g, 92%). Compound **4** was obtained as a colorless oil (8.21 g, 94%).

Compound 3: ^{1}H NMR (400 MHz, CD_3CN) δ 7.79 (d, *J*=8.4, 2H), 7.44 (d, *J*=8.2, 2H), 4.15 – 4.09 (m, 2H), 3.62 – 3.46 (m, 30H), 2.76 (t, *J*=5.8, 5.8, 1H), 2.44 (s, 3H). ^{13}C{^{1}H} NMR (101 MHz, CD_3CN) δ 146.33, 133.80,

130.97, 128.74, 73.28, 71.13, 71.11, 71.09, 71.00, 70.96, 70.93, 69.14, 61.92, 21.60. HRMS (APCI/QTOF) *m/z*: [M + Na]$^+$ calcd. for $C_{23}H_{40}NaO_{11}S^+$ 547.2184, found 547.2181.

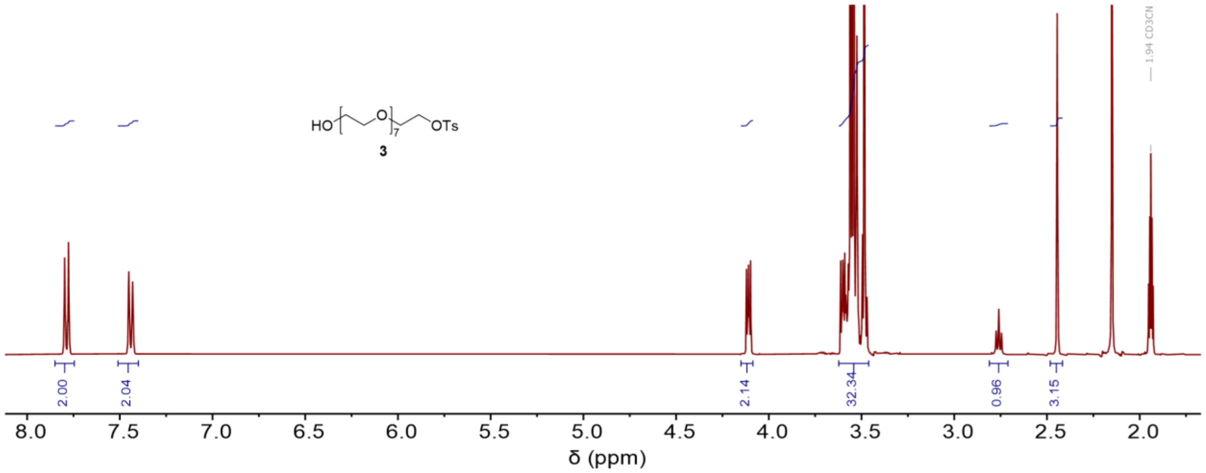

Figure S1. ^{1}H NMR spectrum (400 MHz, CD_3CN) of compound **3**.

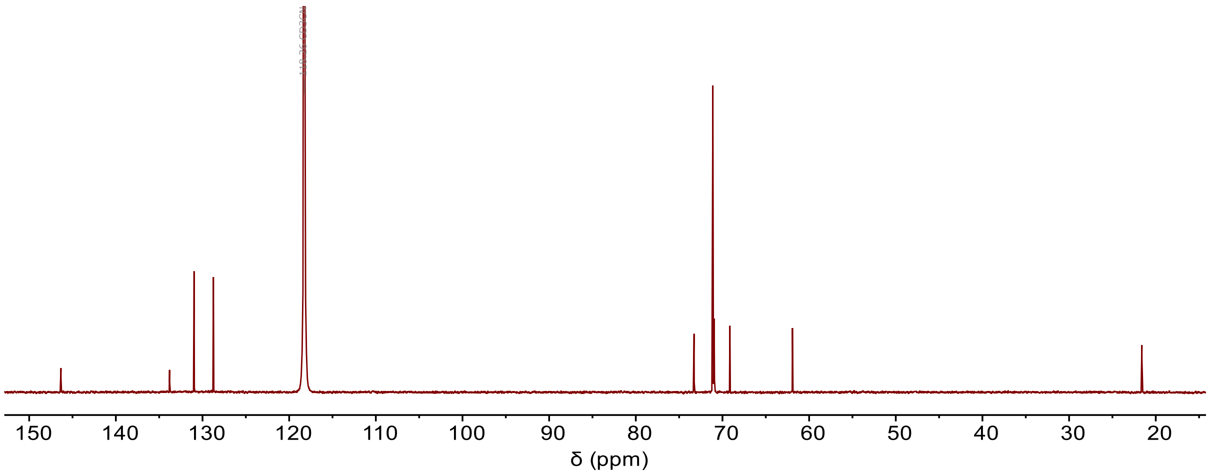

Figure S2. $^{13}C\{^1H\}$ NMR spectrum (101 MHz, CD_3CN) of compound **3**.

Compound 4: ^{1}H NMR (400 MHz, $CDCl_3$) δ 7.81 – 7.76 (d, 2H), 7.47 – 7.41 (dt, 2H), 4.14 – 4.07 (m, 2H), 3.64 – 3.44 (m, 22H), 2.44 (s, 3H). $^{13}C\{^1H\}$ NMR (101 MHz, CD_3CN) δ 146.32, 133.78, 130.96, 128.73, 73.24, 73.22, 71.11, 71.07, 71.05, 71.02, 70.96, 70.94, 70.91, 69.13, 61.92, 21.59. HRMS (ESI/QTOF) *m/z*: [M + Na]$^+$ calcd. for $C_{19}H_{32}NaO_9S^+$ 459.1659, found 459.1676.

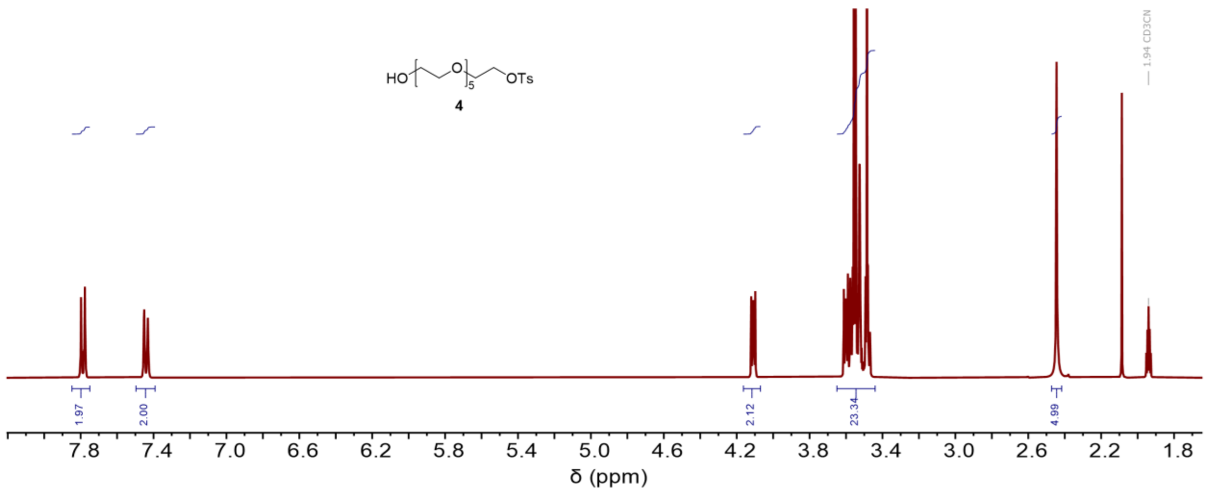

Figure S3. ^{1}H NMR spectrum (400 MHz, CD_3CN) of compound **4**.

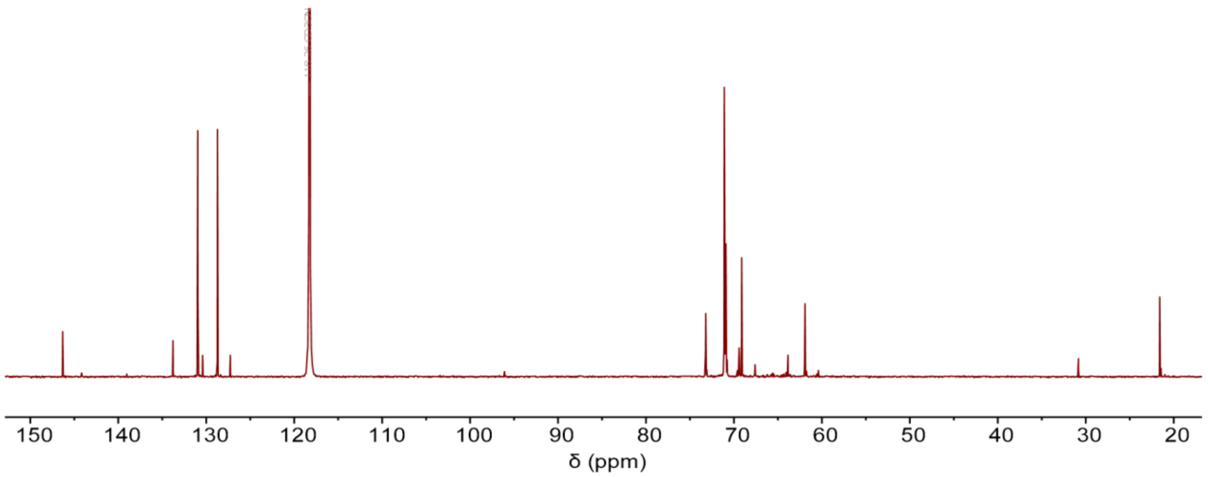

Figure S4. $^{13}C\{^1H\}$ NMR spectrum (101 MHz, CD_3CN) of compound **4**.

Synthesis of compounds 6 and 7

A solution of 3,5-dibromophenol (**5**, 2.29 g, 1.20 equiv, 9.15 mmol) and Cs_2CO_3 (5.00 g, 2.00 equiv, 15.24 mmol) in DMF (30 mL) was stirred at 100 °C for 2 h under a nitrogen atmosphere. Compound **3** or **4** (1.0 equiv, 7.62 mmol) was then added and the resulting mixture was stirred for 24 h at 100˚C. The reaction was quenched with 30 mL of deionized water and the organic layer was extracted with DCM (3 × 60 mL). The organic phases were combined and washed with water (2 × 60 mL) and brine (3 × 60 mL) before being dried over anhydrous magnesium sulfate and concentrated under vacuum. The residue was purified by chromatography (**6**: ethyl acetate: methanol, 10:1; **7**: ethyl acetate: methanol, 15:1). Compound **6** was obtained as a colorless oil (3.73 g, 81%). Compound **7** was obtained as a colorless oil (3.46 g, 88%).

Compound 6: ^{1}H NMR (400 MHz, CD_3CN) δ 7.32 (t, *J*=1.6, 1.6, 1H), 7.13 (d, *J*=1.6, 2H), 4.13 – 4.08 (m, 2H), 3.78 – 3.73 (m, 2H), 3.63 – 3.52 (m, 26H), 3.50 – 3.46 (m, 2H), 2.80 – 2.73 (m, 1H). $^{13}C\{^1H\}$ NMR (101 MHz, CD_3CN) δ 161.32, 127.03, 123.81, 118.11, 73.27, 71.33, 71.15, 71.11, 71.00, 69.91, 69.33, 61.92. HRMS (APCI/QTOF) *m*/*z*: $[M + H]^+$ calcd. for $C_{22}H_{37}Br_2O_9^+$ 603.0799, found 603.0793.

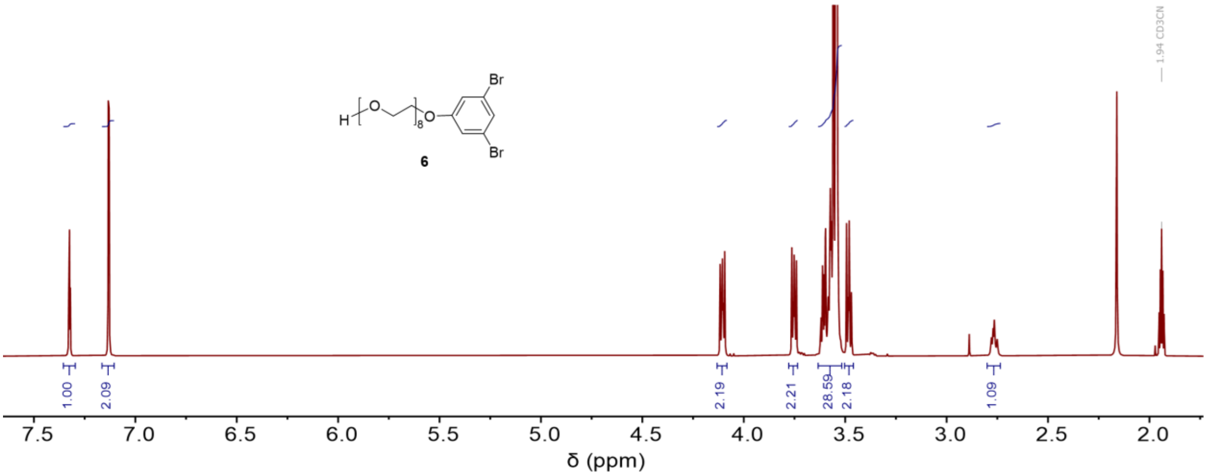

Figure S5. ^{1}H NMR spectrum (400 MHz, CD_3CN) of compound **6**.

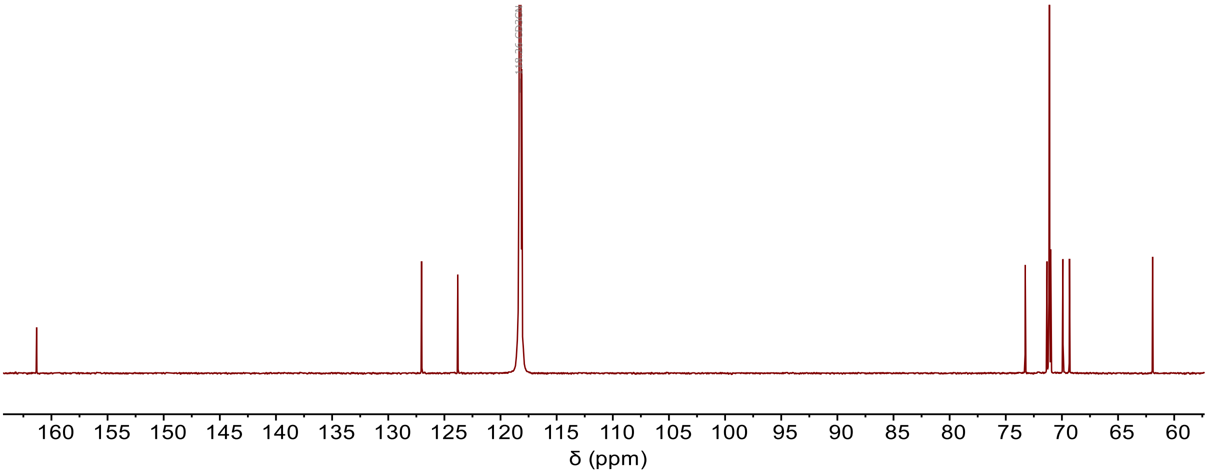

Figure S6. $^{13}C\{^{1}H\}$ NMR spectrum (101 MHz, CD_3CN) of compound **6**.

Compound 7: ^{1}H NMR (400 MHz, CD_3CN) δ 7.32 (q, *J*=1.5, 1.5, 1.4, 1H), 7.12 (t, *J*=1.4, 1.4, 2H), 4.13 – 4.07 (m, 2H), 3.78 – 3.72 (m, 2H), 3.63 – 3.52 (m, 18H), 3.50 – 3.45 (m, 2H), 2.80 (d, *J*=5.5, 1H). $^{13}C\{^{1}H\}$ NMR (101 MHz, CD_3CN) δ 161.29, 127.01, 123.79, 118.08, 73.28, 71.31, 71.14, 71.12, 71.09, 70.98, 69.90, 69.30, 61.92. HRMS (ESI/QTOF) *m*/*z*: $[M + Na]^+$ calcd. for $C_{18}H_{28}Br_2NaO_7^+$ 537.0094, found 537.0114.

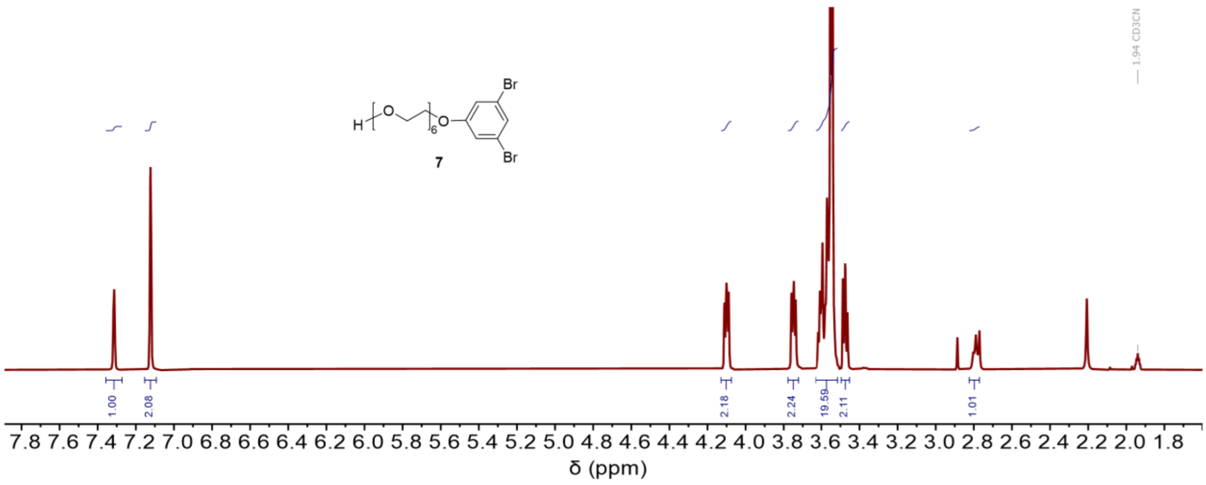

Figure S7. ^{1}H NMR spectrum (400 MHz, CD_3CN) of compound **7**.

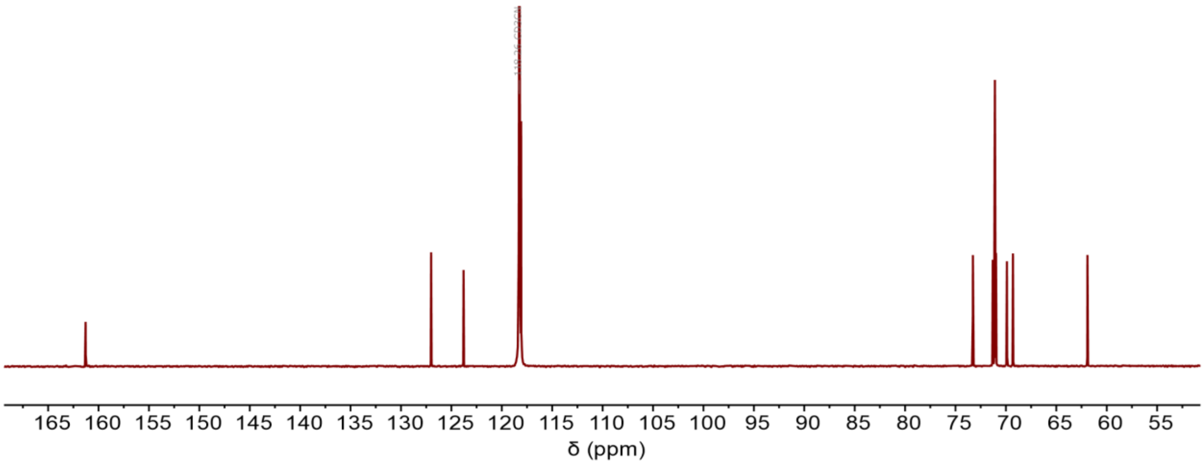

Figure S8. $^{13}C\{^{1}H\}$ NMR spectrum (101 MHz, CD_3CN) of compound **7**.

Synthesis of compound 10

Compound **6** (1.81 g, 1.0 equiv, 3.0 mmol), 3-pyridineboronic acid pinacol ester (**8**) (1.48 g, 2.4 equiv, 7.2 mmol), $Pd(PPh_3)_4$ (0.35 g, 0.1 equiv, 0.3 mmol), and K_2CO_3 (4.14 g, 10 equiv, 30 mmol) were added into a 50 mL single-neck round-bottom flask equipped with a magnetic stir bar and capped with a septum. Under a nitrogen atmosphere, DMF (20 mL) along with H_2O (1 mL) were added and the resulting mixture was stirred for 60 h at 100 °C. After cooling to room temperature, the mixture was diluted with chloroform/methanol (10/1, v/v) and filtered through Celite. The filtrate was washed with brine, dried over anhydrous magnesium sulfate, and purified by column chromatography (methanol: ethyl acetate, 1:3). Compound **10** was obtained as a colorless oil (1.42 g, 79%).

^{1}H NMR (400 MHz, CD_3CN) δ 8.94 (d, *J*=1.8, 2H), 8.59 (dd, *J*=4.8, 1.6, 2H), 8.08 (dt, *J*=7.9, 1.7, 1.7, 2H), 7.54 (t, *J*=1.5, 1.5, 1H), 7.44 (ddd, *J*=7.9, 4.8, 0.7, 2H), 7.27 (d, *J*=1.5, 2H), 4.28 (dd, *J*=5.4, 3.8, 2H), 3.94 – 3.79 (m, 2H), 3.65 (dt, *J*=3.9, 2.4, 2.4, 2H), 3.62 – 3.45 (m, 26H), 2.84 (t, *J*=5.7, 5.7, 1H). $^{13}C\{^{1}H\}$ NMR (101 MHz, CD_3CN) δ 160.99, 149.84, 149.21, 140.98, 136.73, 135.48, 124.60, 119.45, 113.90, 73.29, 71.33, 71.13, 71.09,

71.07, 71.05, 70.95, 70.26, 68.82, 61.88. HRMS (APCI/QTOF) *m*/*z*: $[M + Na]^+$ calcd. for $C_{32}H_{44}N_2NaO_9^+$ 623.2939, found 623.2942.

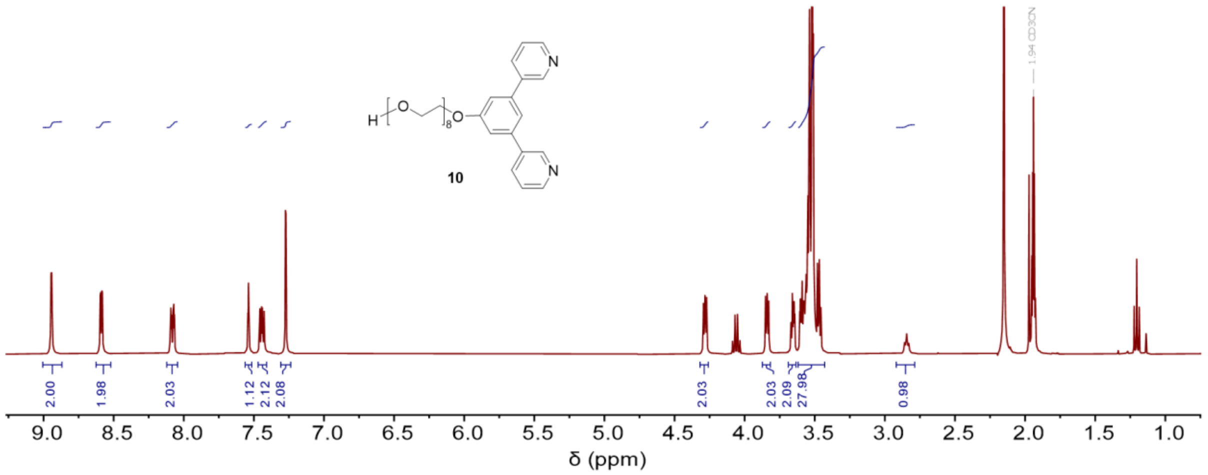

Figure S9. ^1H NMR spectrum (400 MHz, CD_3CN) of compound **10**.

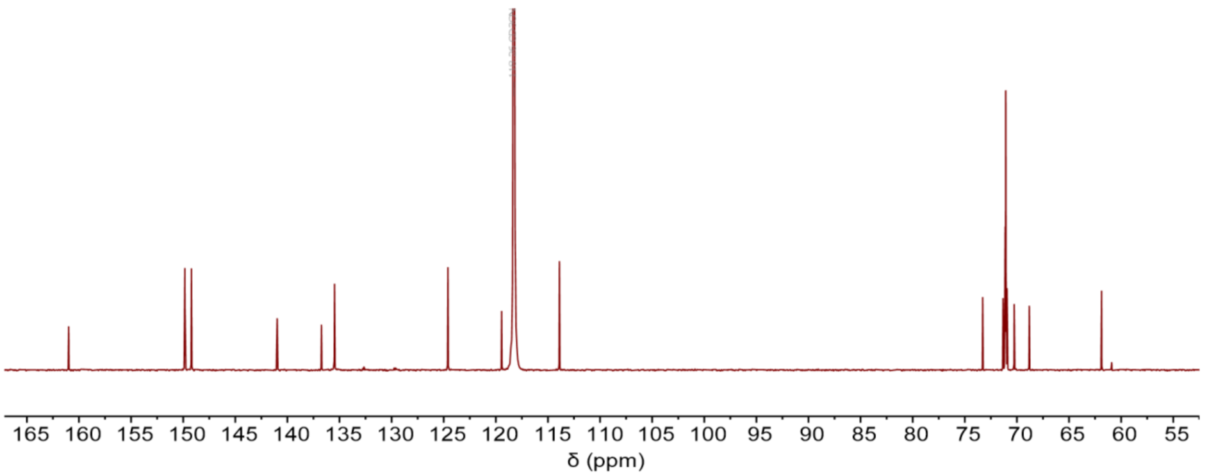

Figure S10. $^{13}C\{^1H\}$ NMR spectrum (101 MHz, CD_3CN) of compound **10**.

Synthesis of compound 11

Compound **11** was synthesized following a similar procedure as used for compound **10**. Specifically, compound **7** (1.28 g, 1.0 equiv, 3.0 mmol), 4-pyridineboronic acid pinacol ester (**9**) (1.48 g, 2.4 equiv, 7.2 mmol), $Pd(PPh_3)_4$ (0.35 g, 0.1 equiv, 0.3 mmol), and K_2CO_3 (4.14 g, 10 equiv, 30 mmol) were added into a 50 mL single-neck round-bottom flask equipped with a magnetic stir bar and capped with a septum. Under a nitrogen atmosphere, DMF (20 mL) along with H_2O (1 mL) were added and the resulting mixture was stirred for 60 h at 100 °C. After cooling to room temperature, the mixture was diluted with chloroform/methanol (10/1, v/v) and filtered through Celite. The filtrate was washed with brine, dried over anhydrous magnesium sulfate, and purified by column chromatography (methanol: ethyl acetate, 1:3). Compound **11** was obtained as a colorless oil (1.32 g, 86%).

^{1}H NMR (400 MHz, CD_3CN) δ 8.70 – 8.59 (m, 4H), 7.71 – 7.63 (m, 4H), 7.60 (t, *J*=1.5, 1.5, 1H), 7.32 (d, *J*=1.5, 2H), 4.30 – 4.22 (m, 2H), 3.86 – 3.79 (m, 2H), 3.67 – 3.61 (m, 2H), 3.61 – 3.48 (m, 16H), 3.45 (dd, *J*=5.5, 4.1, 2H), 3.13 (s, 1H). $^{13}C\{^{1}H\}$ NMR (101 MHz, CD_3CN) δ 160.99, 151.16, 148.16, 141.15, 122.67, 119.13, 114.72, 73.30, 71.31, 71.10, 71.07, 71.05, 71.04, 70.92, 70.21, 68.86, 61.86. HRMS (ESI/QTOF) *m*/*z*: $[M + H]^{+}$ calcd. for $C_{28}H_{37}N_2O_7^{+}$ 513.2595, found 513.2606.

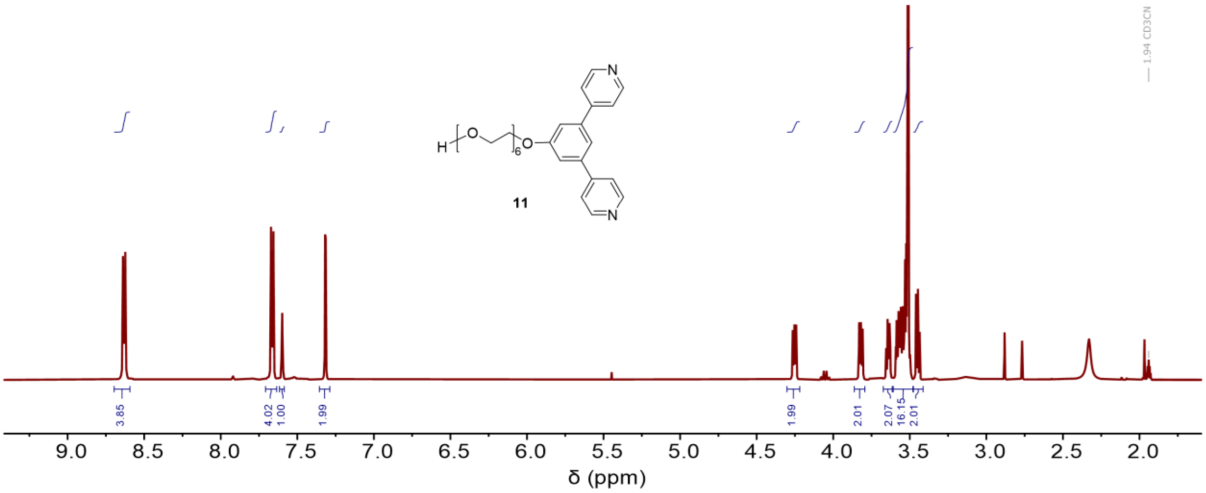

Figure S11. ^{1}H NMR spectrum (400 MHz, CD_3CN) of compound **11**.

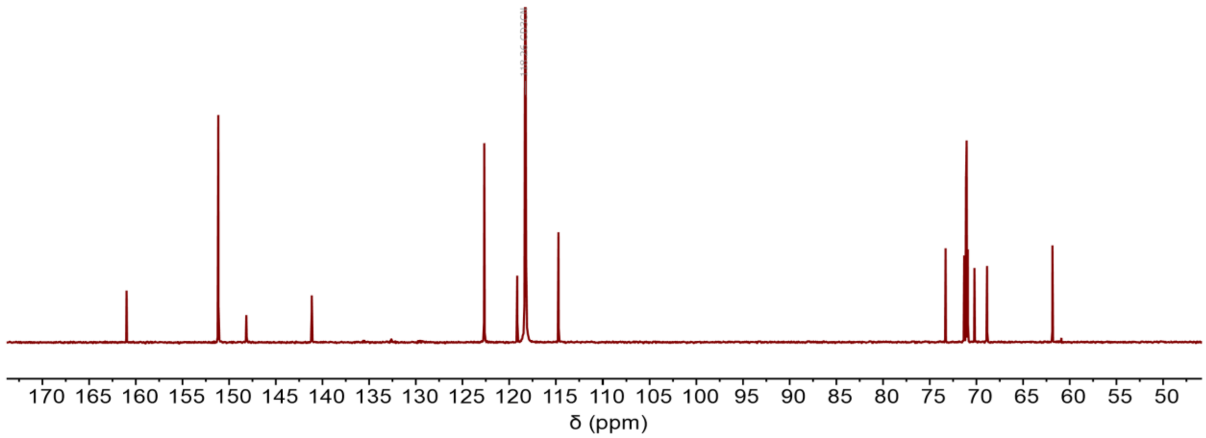

Figure S12. $^{13}C\{^{1}H\}$ NMR spectrum (101 MHz, CD_3CN) of compound **11**.

Synthesis of compounds L1 and L2

Compound **10** or **11** (1.0 equiv, 2.0 mmol) and triethylamine (1.15 mL, 4.0 equiv, 8.0 mmol) were dissolved in DCM (25 mL) and the resulting mixture was stirred at 0 °C for 1 h under a nitrogen atmosphere. Subsequently, acryloyl chloride (0.24 mL, 1.5 equiv, 3.0 mmol) was added. The solution was stirred at 0 °C for 3 h. After that, the solution was allowed to stand at room temperature for 36 h. The solution was then concentrated under vacuum and the residue was purified by chromatography on silica (**L1**: hexane: ethyl acetate, 1:20; **L2**: hexane: ethyl acetate, 1:30). Compound **L1** was obtained as a yellowish oil (1.14 g, 87%). Compound **L2** was obtained as a yellowish oil (1.03 g, 91%).

Compound L1: ^{1}H NMR (400 MHz, CD_3CN) δ 8.93 (dd, *J*=2.5, 0.9, 2H), 8.58 (dd, *J*=4.8, 1.6, 2H), 8.06 (ddd, *J*=7.9, 2.4, 1.6, 2H), 7.52 (t, *J*=1.5, 1.5, 1H), 7.43 (ddd, *J*=7.9, 4.8, 0.9, 2H), 7.25 (d, *J*=1.5, 2H), 6.34 (dd, *J*=17.3,

1.5, 1H), 6.13 (dd, *J*=17.3, 10.4, 1H), 5.85 (dd, *J*=10.4, 1.5, 1H), 4.28 – 4.24 (m, 2H), 4.24 – 4.20 (m, 2H), 3.86 – 3.79 (m, 2H), 3.67 – 3.62 (m, 4H), 3.61 – 3.47 (m, 22H). $^{13}C\{^{1}H\}$ NMR (101 MHz, CD_3CN) δ 166.73, 160.96, 149.80, 149.17, 140.94, 136.70, 135.45, 131.60, 129.21, 124.59, 119.41, 113.87, 71.32, 71.12, 71.06, 71.05, 71.04, 71.00, 70.26, 69.54, 68.79, 64.52. HRMS (ESI/QTOF) *m*/*z*: $[M + H]^+$ calcd. for $C_{31}H_{39}N_2O_8^+$ 567.2701, found 567.2711.

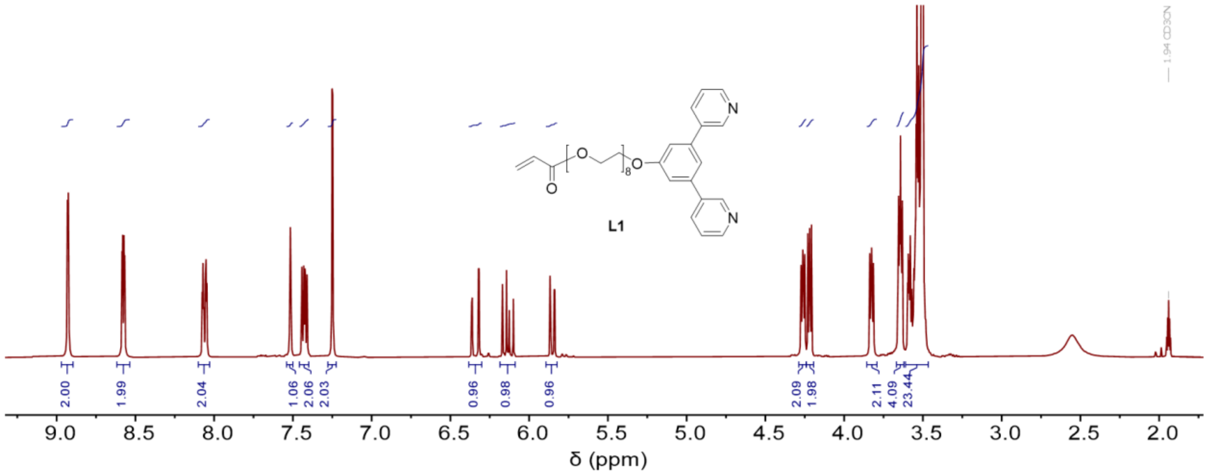

Figure S13. ^{1}H NMR spectrum (400 MHz, CD_3CN) of compound **L1**.

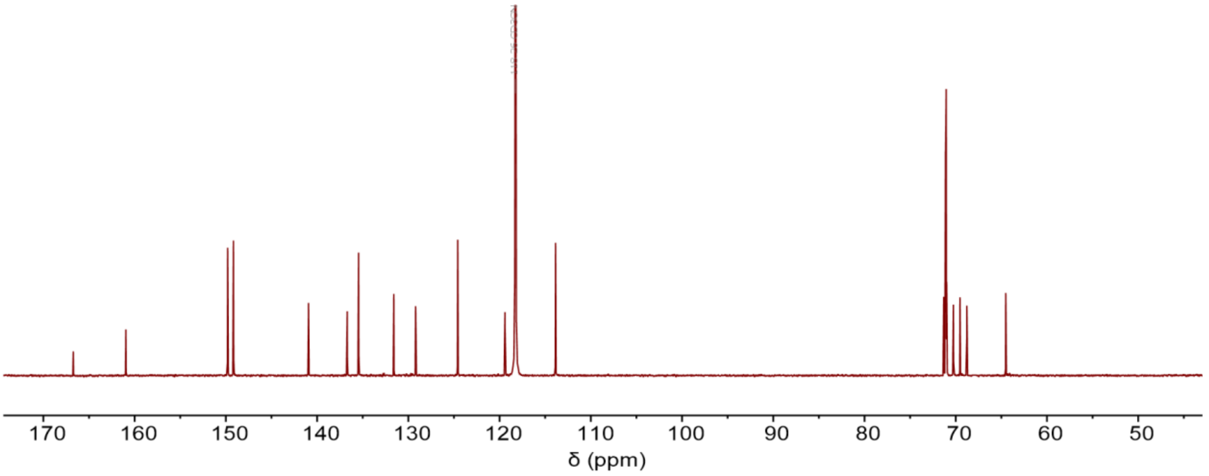

Figure S14. $^{13}C\{^{1}H\}$ NMR spectrum (101 MHz, CD_3CN) of compound **L1**.

Compound L2: ^{1}H NMR (400 MHz, CD_3CN) δ 8.68 – 8.62 (m, 4H), 7.73 – 7.68 (m, 4H), 7.64 (t, *J*=1.6, 1.6, 1H), 7.36 (d, *J*=1.5, 2H), 6.34 (dd, *J*=17.3, 1.6, 1H), 6.13 (dd, *J*=17.3, 10.4, 1H), 5.85 (dd, *J*=10.4, 1.5, 1H), 4.32 – 4.26 (m, 2H), 4.24 – 4.19 (m, 2H), 3.87 – 3.82 (m, 2H), 3.68 – 3.61 (m, 4H), 3.61 – 3.57 (m, 2H), 3.57 – 3.49 (m, 12H). $^{13}C\{^{1}H\}$ NMR (101 MHz, CD_3CN) δ 166.75, 161.07, 151.23, 148.19, 141.31, 131.58, 129.22, 122.71, 119.23, 118.26, 114.77, 71.35, 71.14, 71.13, 71.11, 71.09, 71.07, 71.01, 70.25, 69.55, 68.93, 64.53. HRMS (APCI/QTOF) *m*/*z*: $[M + Na]^+$ calcd. for $C_{35}H_{46}N_2NaO_{10}^+$ 677.3045, found 677.3039.

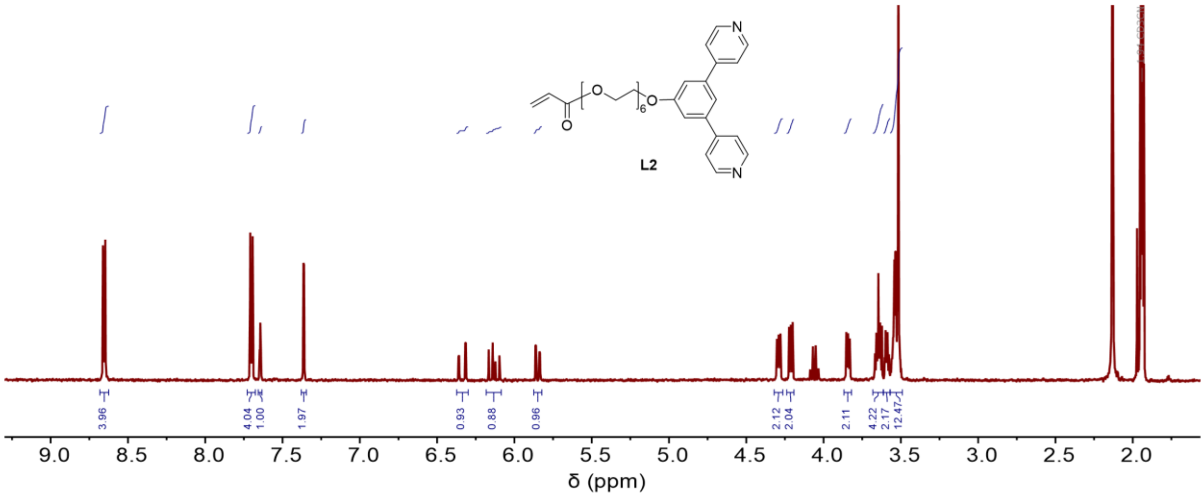

Figure S15. ^{1}H NMR spectrum (400 MHz, CD_3CN) of compound **L2.**

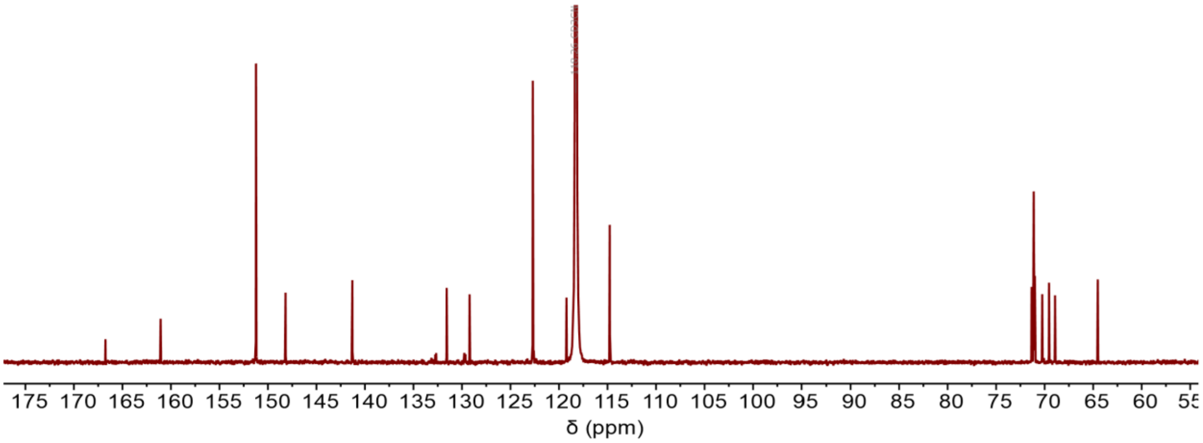

Figure S16. $^{13}C\{^{1}H\}$ NMR spectrum (101 MHz, CD_3CN) of compound **L2**.

2.2 Synthesis of hydrogel HG0 by Pd^{2+}-mediated crosslinking of a polymeric ligand

The hydrogel **HG0** was synthesized according to a modified literature procedure.[1] Specifically, 20 mg of the polymeric ligand **L1'-PEG$_{4600}$-L1'** was dissolved in 460 μL of D_2O. Subsequently,40 μL of a 100 mM $Pd(NO_3)_2$ stock solution in D_2O was added. The mixture was vortexed till they were mixed well before it was annealed at 70 °C for 4 h to obtain hydrogel **HG0**.

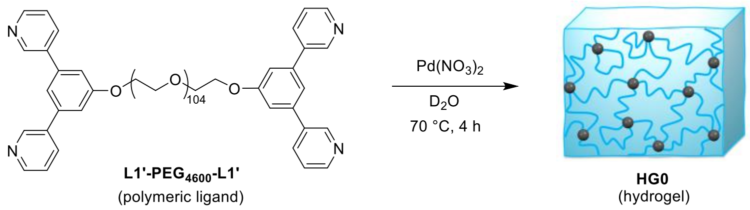

Scheme S3. Synthesis of supramolecular hydrogel **HG0**.

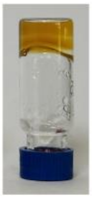

HG0

Figure S17. Picture of the supramolecular hydrogel **HG0**. The strong coloration indicates partial decomposition of the Pd complexes.

2.3 Synthesis and characterization of cage $[Pd_2(L1)_4](NO_3)_4$

Cage $[Pd_2(\mathbf{L1})_4](NO_3)_4$ was synthesized by stirring a mixture of ligand **L1** (50 µmol, 1000 µL of a 50 mM stock solution of **L1** in CD_3CN) and $Pd(NO_3)_2$ (25 µmol, 500 µL of a 50 mM stock solution in CD_3CN) in CD_3CN (1000 µL) at 80 °C for 2 h to give 2500 µL of a 5 mM solution of $[Pd_2(\mathbf{L1})_4](NO_3)_4$. The cage was characterized by NMR spectroscopy and mass spectrometry. Subsequently, the solvent was removed under reduced pressure and the residue was dissolved in D_2O (2500 µL) to give a 5 mM solution of $[Pd_2(\mathbf{L1})_4](NO_3)_4$. The structural integrity of the cage after solvent exchange was verified by ^{1}H NMR spectroscopy.

^{1}H NMR (400 MHz, CD_3CN) δ 10.26 (d, *J*=2.0, 2H), 9.49 (dd, *J*=5.8, 1.3, 2H), 8.79 (t, *J*=1.6, 1.6, 1H), 8.30 (dt, *J*=8.3, 1.6, 1.6, 2H), 7.66 (dd, *J*=8.1, 5.7, 2H), 7.24 (d, *J*=1.5, 2H), 6.28 (dd, *J*=17.3, 1.5, 1H), 6.07 (dd, *J*=17.3, 10.4, 1H), 5.80 (dd, *J*=10.4, 1.5, 1H), 4.19 – 4.10 (m, 4H), 3.76 – 3.69 (m, 2H), 3.60 – 3.54 (m, 4H), 3.51 – 3.39 (m, 22H). ^{13}C{^{1}H} NMR (126 MHz, CD_3CN) δ 166.66, 161.48, 151.49, 151.35, 139.96, 139.41, 138.02, 131.58, 129.14, 128.16, 120.18, 114.98, 71.25, 71.08, 71.04, 71.02, 70.99, 70.98, 70.96, 70.07, 69.50, 68.99, 64.47. HRMS (APCI/QTOF) *m/z*: $[M - 3(NO_3)]^{3+}$ calcd. for $Pd_2C_{140}H_{184}N_9O_{43}{}^{3+}$ most abundant 964.35412, found 964.35407.

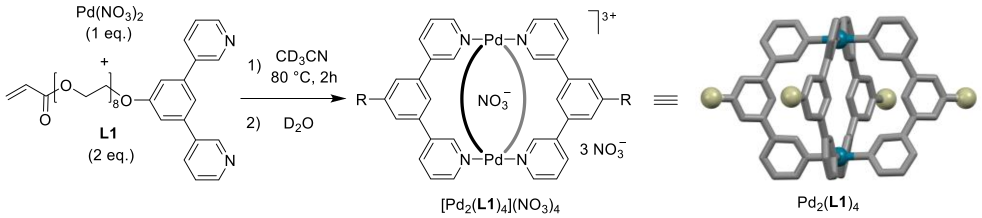

Scheme S4. Synthesis of cage $[Pd_2(\mathbf{L1})_4](NO_3)_4$.

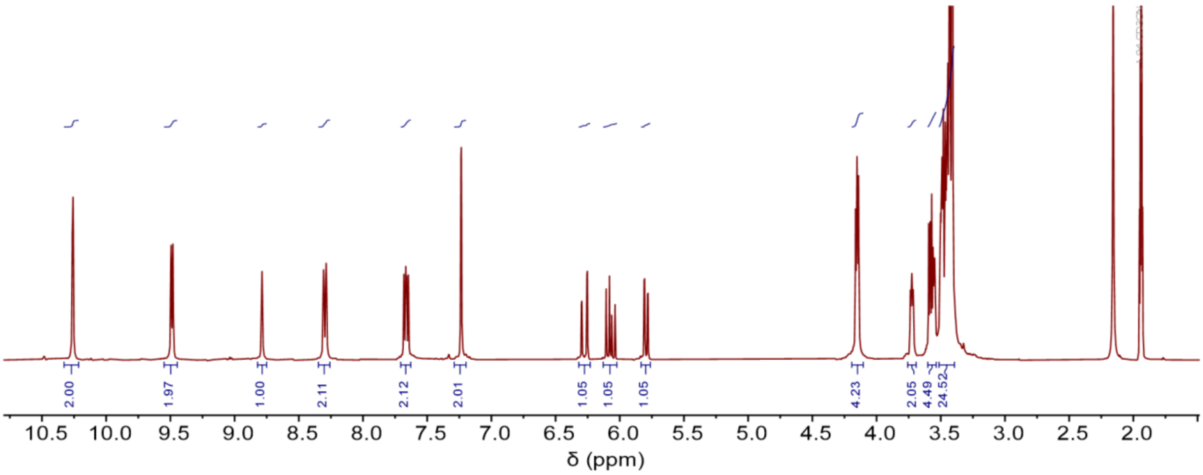

Figure S18. ^{1}H NMR spectrum (400 MHz, CD_3CN) of cage $[Pd_2(\mathbf{L1})_4](NO_3)_4$.

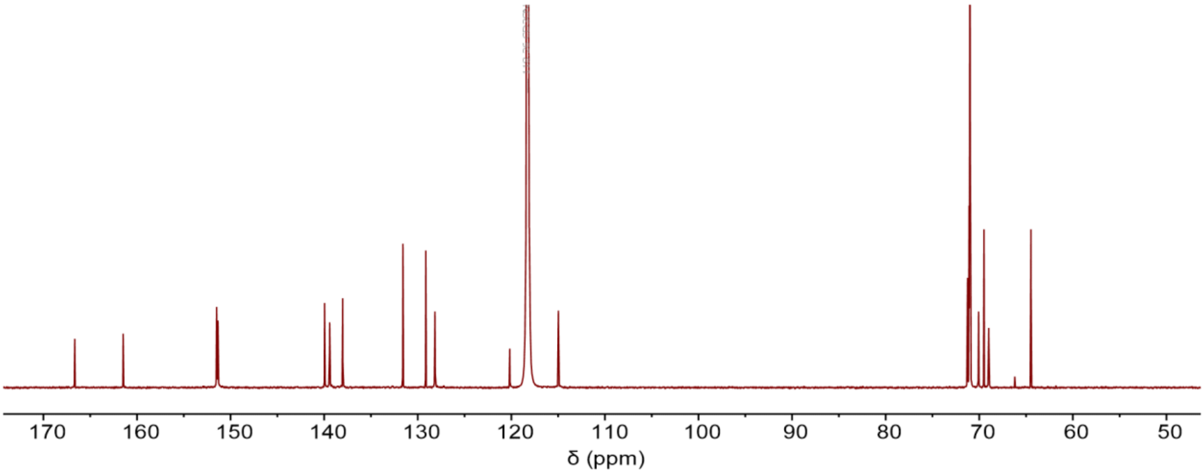

Figure S19. $^{13}C\{^{1}H\}$ NMR spectrum (101 MHz, CD_3CN) of cage $[Pd_2(\mathbf{L1})_4](NO_3)_4$.

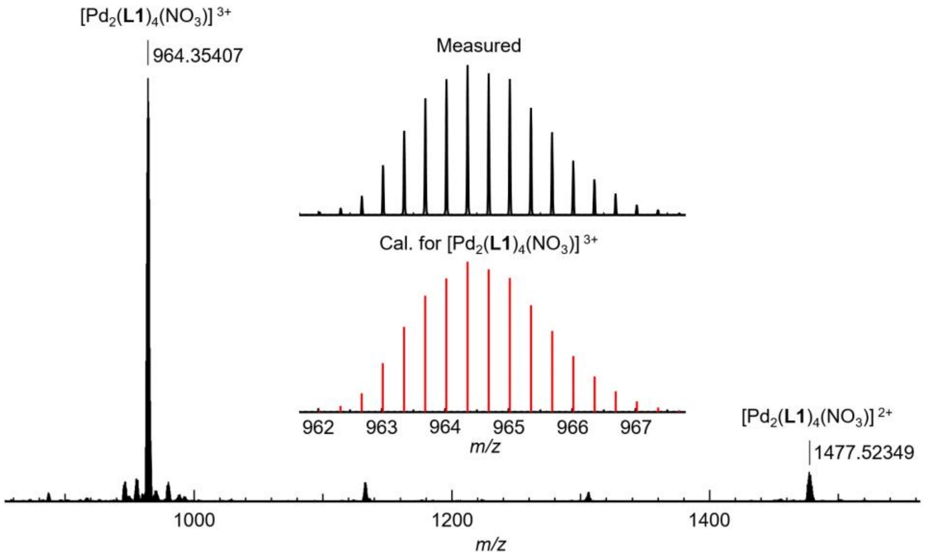

Figure S20. High-resolution ESI-MS spectrum of cage $[Pd_2(\mathbf{L1})_4](NO_3)_4$.

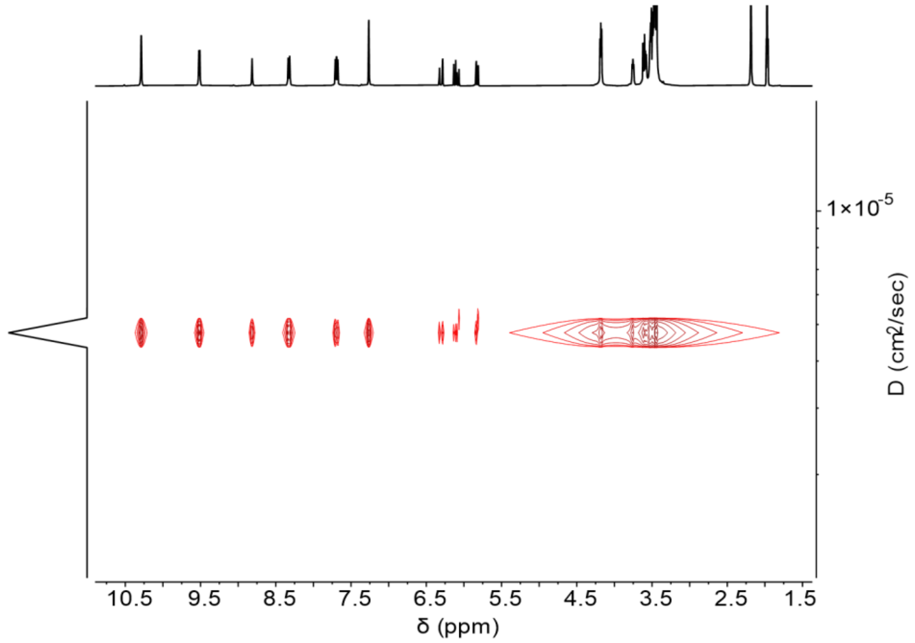

Figure S21. DOSY NMR spectrum of cage [Pd$_2$(**L1**)$_4$](NO$_3$)$_4$.

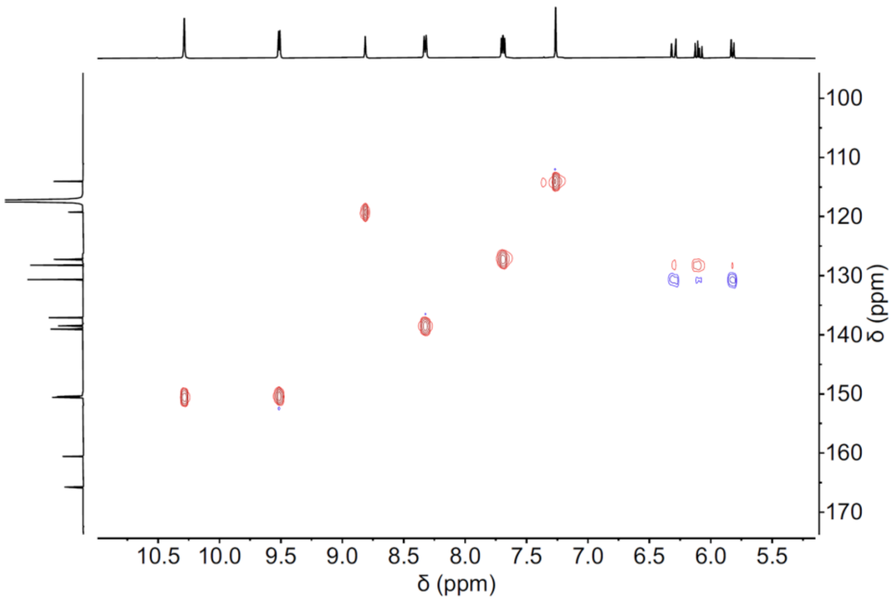

Figure S22. HSQC NMR spectrum of cage [Pd$_2$(**L1**)$_4$](NO$_3$)$_4$.

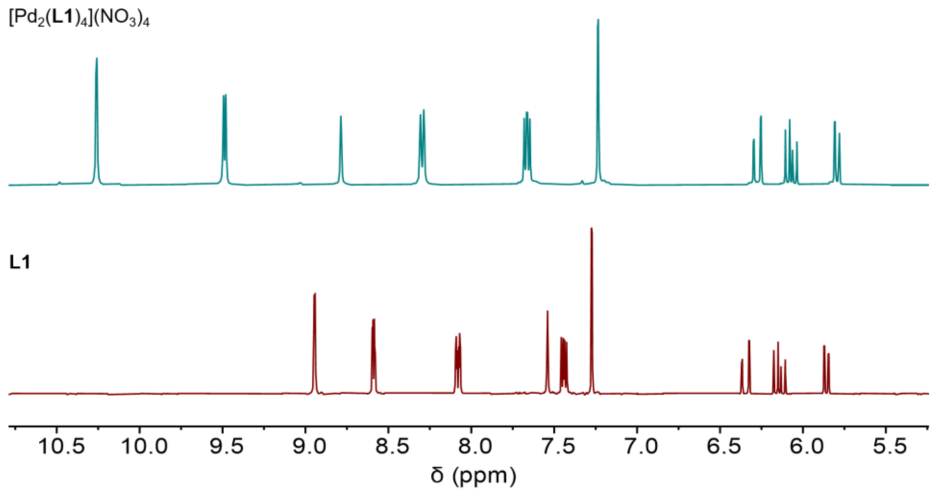

Figure S23. ^{1}H NMR spectrum (400 MHz, CD_3CN) of cage [Pd_2(**L1**)$_4$](NO_3)$_4$ and **L1**.

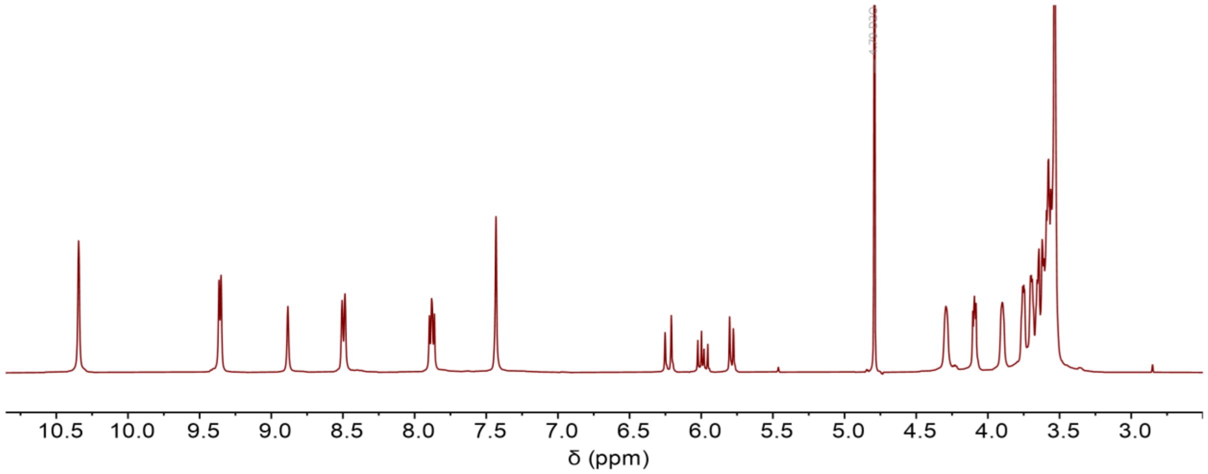

Figure S24. ^{1}H NMR spectrum (400 MHz, D_2O) of cage [Pd_2(**L1**)$_4$](NO_3)$_4$.

2.4 Synthesis and characterization of cage [Pd_4(L1)$_8$] (BF_4)$_8$

Cage [Pd_4(**L1**)$_8$](BF_4)$_8$ was synthesized by stirring a mixture of ligand **L1** (25 µmol, 500 µL of a 50 mM stock solution of **L1** in CD_3CN) and [Pd(CH_3CN)$_4$](BF_4)$_2$ (12.5 µmol, 250 µL of a 50 mM stock solution in CD_3CN) in CD_3CN (500 µL) at 80 °C for 2 h. Subsequently, the solvent was removed under reduced pressure and the residue was dissolved in D_2O (1250 µL) to give a 2.5 mM solution of [Pd_2(**L1**)$_8$] (BF_4)$_8$. ^{1}H NMR (500 MHz, D_2O) δ 10.45 (s, 1H), 10.20 (s, 1H), 9.84 (dd, *J* = 10.2, 5.7 Hz, 3H), 9.69 (s, 1H), 9.49 (d, *J* = 24.2 Hz, 3H), 9.29 – 9.16 (m, 3H), 9.10 (d, *J* = 5.5 Hz, 1H), 8.70 (d, *J* = 5.5 Hz, 1H), 8.61 (d, *J* = 8.1 Hz, 1H), 8.53 (d, *J* = 8.3 Hz, 1H), 8.41 (dd, *J* = 21.6, 8.2 Hz, 3H), 8.30 (d, *J* = 8.1 Hz, 1H), 8.23 (s, 1H), 8.18 (dd, *J* = 11.5, 7.0 Hz, 2H), 8.11 (s, 1H), 7.98 (p, *J* = 5.9 Hz, 3H), 7.87 (s, 1H), 7.86 – 7.79 (m, 2H), 7.78 – 7.70 (m, 3H), 7.66 (s, 1H), 7.62 – 7.56 (m, 1H), 7.42 (s, 1H), 7.40 (s, 1H), 7.33 (s, 2H), 7.28 (s, 1H), 6.94 (s, 1H), 6.89 (s, 1H), 6.49 (s, 1H), 6.12 – 5.98 (m, 5H), 5.93 – 5.78 (m, 4H), 5.72 – 5.57 (m, 4H), 4.40 (s, 2H), 4.24 (d, *J* = 7.7 Hz, 5H), 4.04 (s, 3H), 4.00 – 3.93 (m, 7H), 3.92 – 3.81 (m, 10H), 3.76 (t, *J* = 4.4 Hz, 3H), 3.73 – 3.32 (m, 127H), 3.02 (d, *J* = 31.5 Hz, 2H), 2.91 – 2.76 (m, 2H). HRMS (APCI/QTOF) *m*/*z*: [M - 3(NO_3)]$^{3+}$ calcd. for $Pd_4C_{280}H_{368}N_{16}O_{80}B_3F_{12}^{5+}$ most abundant 1184.62928, found 1184.62919.

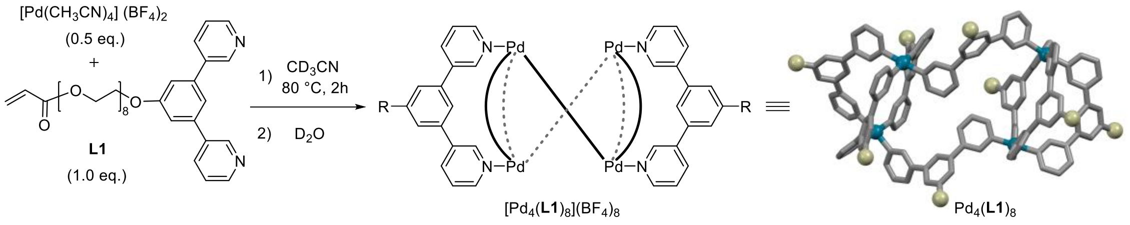

Scheme S5. Synthesis of cage $[Pd_4(\mathbf{L1})_8](BF_4)_8$.

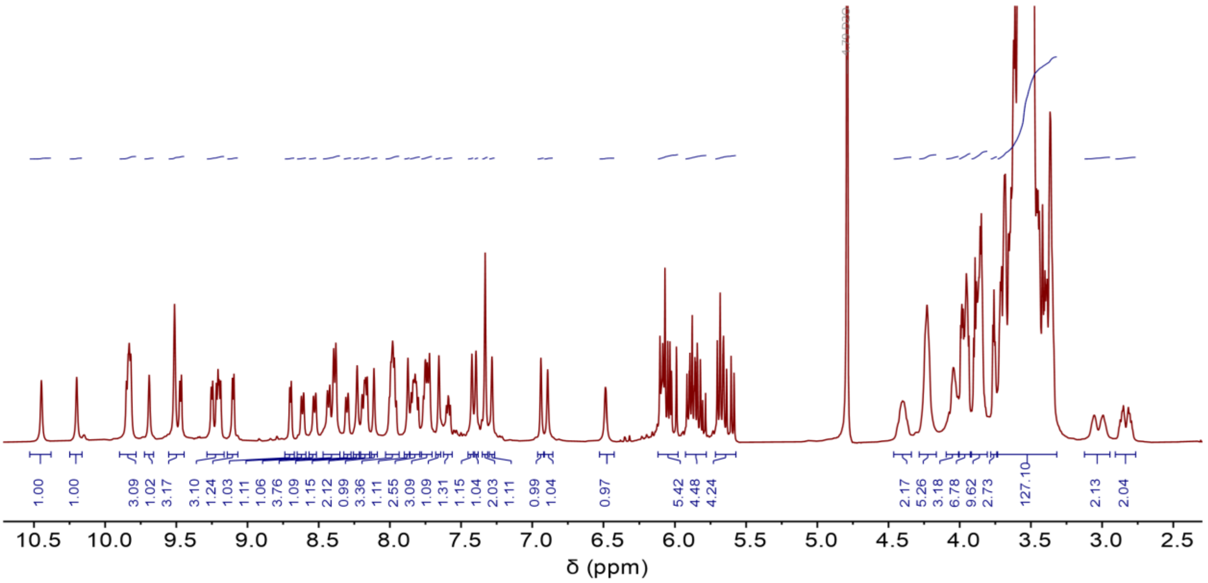

Figure S25. ^{1}H NMR spectrum (500 MHz, D_2O) of cage $[Pd_4(\mathbf{L1})_8](BF_4)_8$.

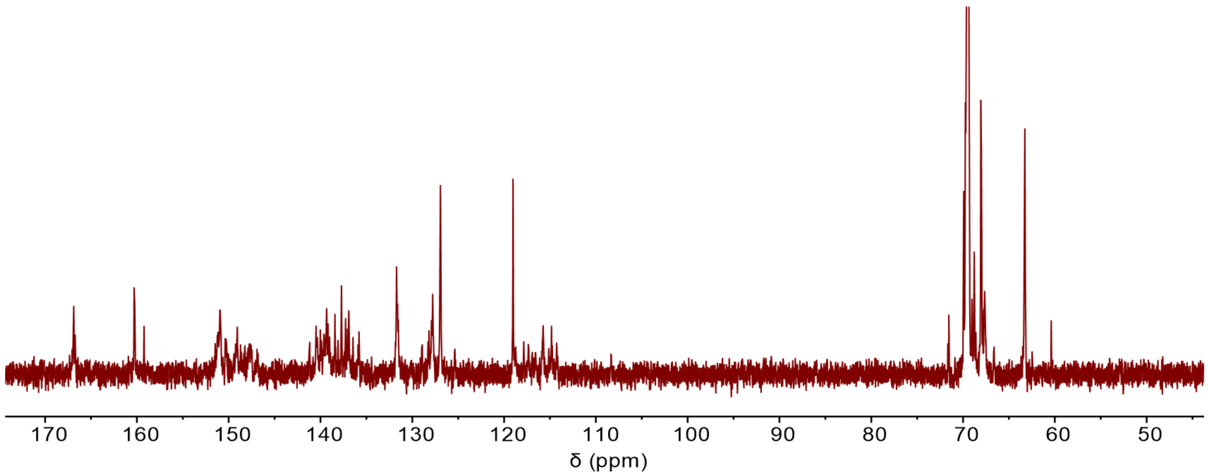

Figure S26. $^{13}C\{^1H\}$ NMR spectrum (151 MHz, D_2O) of cage $[Pd_4(\mathbf{L1})_8](BF_4)_8$. The poor quality of the spectrum is related to the low apparent symmetry of the cage and its limited solubility in D_2O.

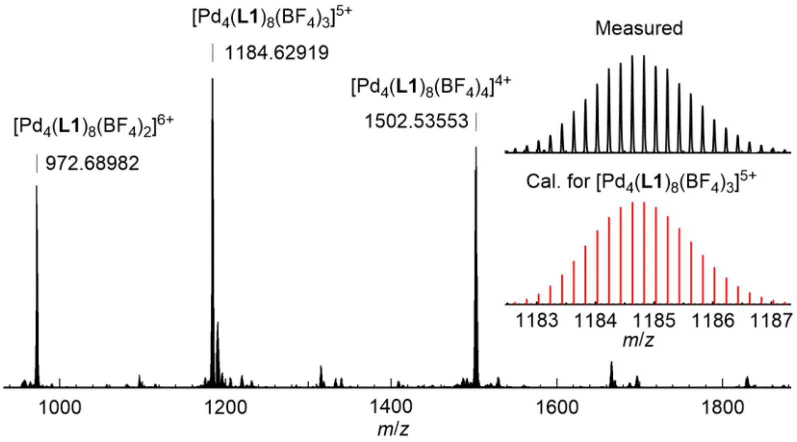

Figure S27. High-resolution ESI-MS spectrum of cage $[Pd_4(\mathbf{L1})_8](BF_4)_8$.

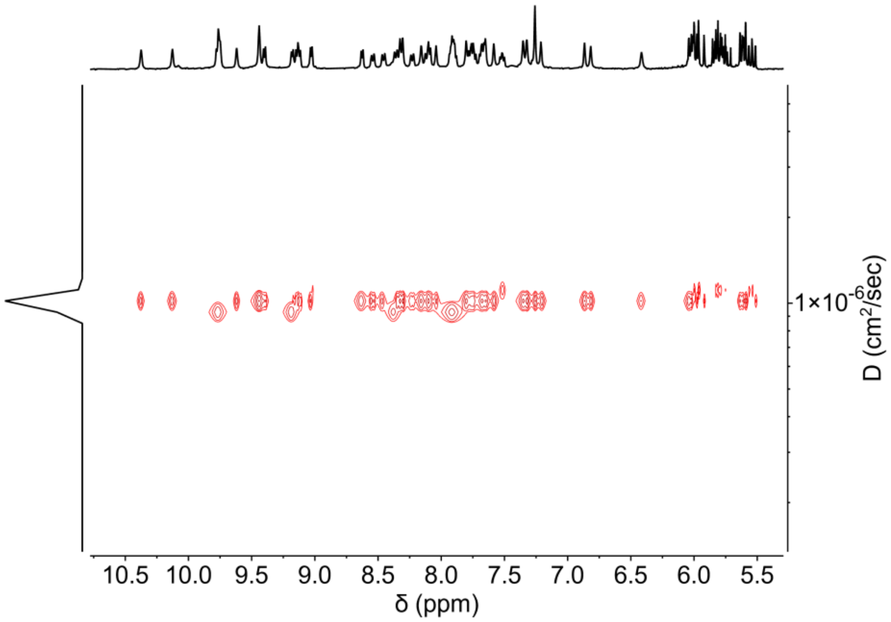

Figure S28. DOSY NMR spectrum of cage $[Pd_4(\mathbf{L1})_8](BF_4)_8$.

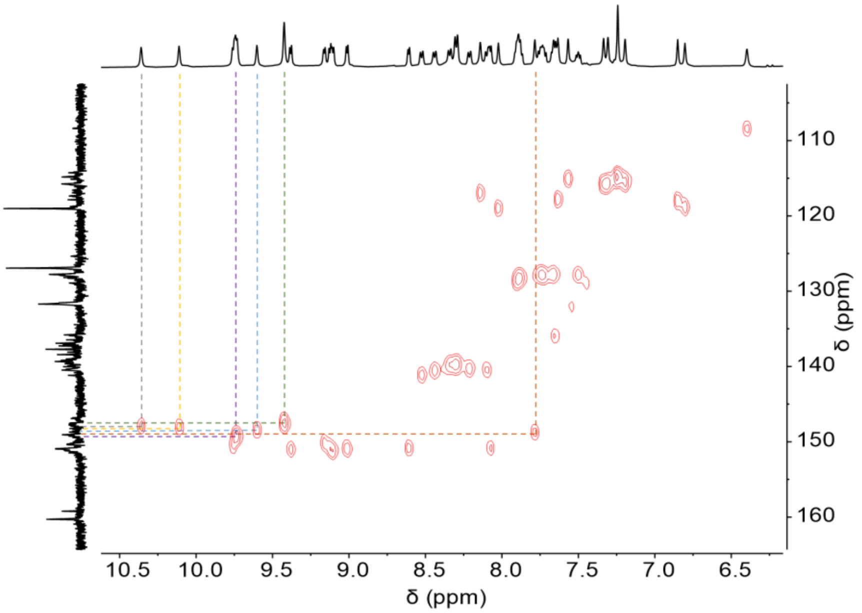

Figure S29. HSQC NMR spectrum of cage [Pd_4(**L1**)$_8$](BF_4)$_8$.

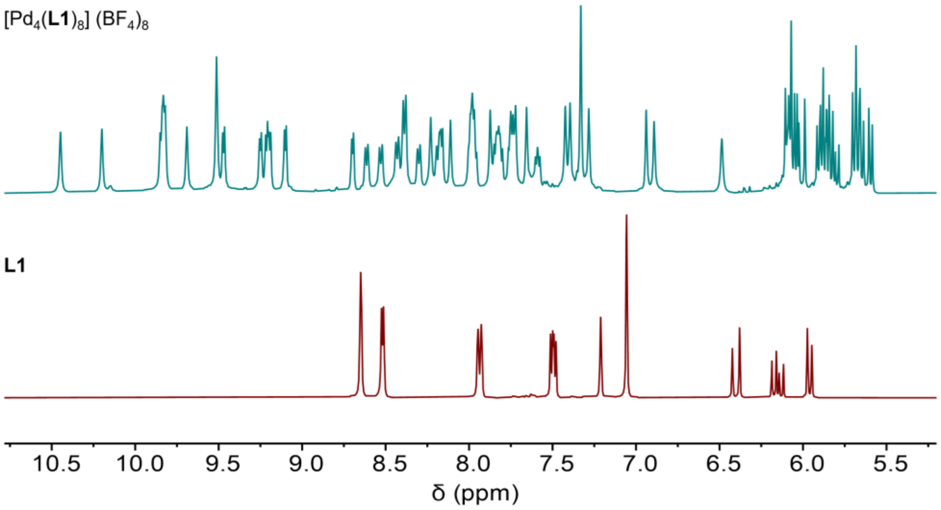

Figure S30. ^{1}H NMR spectrum (400 MHz, D_2O) of cage [Pd_4(**L1**)$_8$](BF_4)$_8$ and **L1**.

2.5 Synthesis and characterization of cage [Pd_{12}(L2)$_{24}$](NO_3)$_{24}$

Cage [Pd_{12}(**L2**)$_{24}$](NO_3)$_{24}$ was synthesized by stirring a mixture of ligand **L2** (25 µmol, 500 µL of a 50 mM stock solution of **L2** in DMSO-d$_6$) and Pd(NO_3)$_2$ (12.5 µmol, 250 µL of a 50 mM stock solution in DMSO-d$_6$) in DMSO-d$_6$ (500 µL) at 80 °C for 6 h to give 1250 µL of a 0.83 mM solution of [Pd_{12}(**L2**)$_{24}$](NO_3)$_{24}$. The cage was characterized by NMR spectroscopy. Attempts to obtain a mass spectrum of the cage were not successful. Subsequently, the solution was diluted five times with D_2O and then lyophilized. The residue was dissolved in D_2O (1250 µL) to give 1250 µL of a 0.83 mM solution of [Pd_{12}(**L2**)$_{24}$](NO_3)$_{24}$. ^{1}H NMR (400 MHz, DMSO-d$_6$) δ 9.64 – 9.38 (m, 4H), 8.30 (d, J = 5.9 Hz, 4H), 8.02 (s, 1H), 7.55 (s, 2H), 6.20 (dd, J = 17.3, 1.6 Hz, 1H), 6.02 (dd, J = 17.3, 10.3 Hz, 1H), 5.76 (dd, J = 10.4, 1.6 Hz, 1H), 4.22 (s, 2H), 4.18 – 3.98 (m, 2H), 3.75 (s, 2H), 3.69 – 3.31

(m, 18H).^{13}C{^{1}H} NMR (151 MHz, DMSO) δ 171.51, 165.31, 159.96, 151.05, 149.16, 136.30, 131.48, 128.02, 124.39, 118.28, 115.38, 69.89, 69.76, 69.72, 69.69, 69.66, 69.63, 68.77, 68.11, 67.76, 63.31.

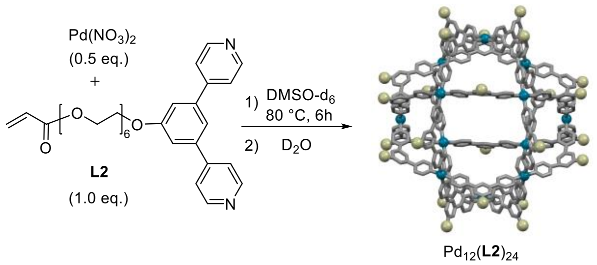

Scheme S6. Synthesis of cage $[Pd_{12}(\mathbf{L2})_{24}](NO_3)_{24}$.

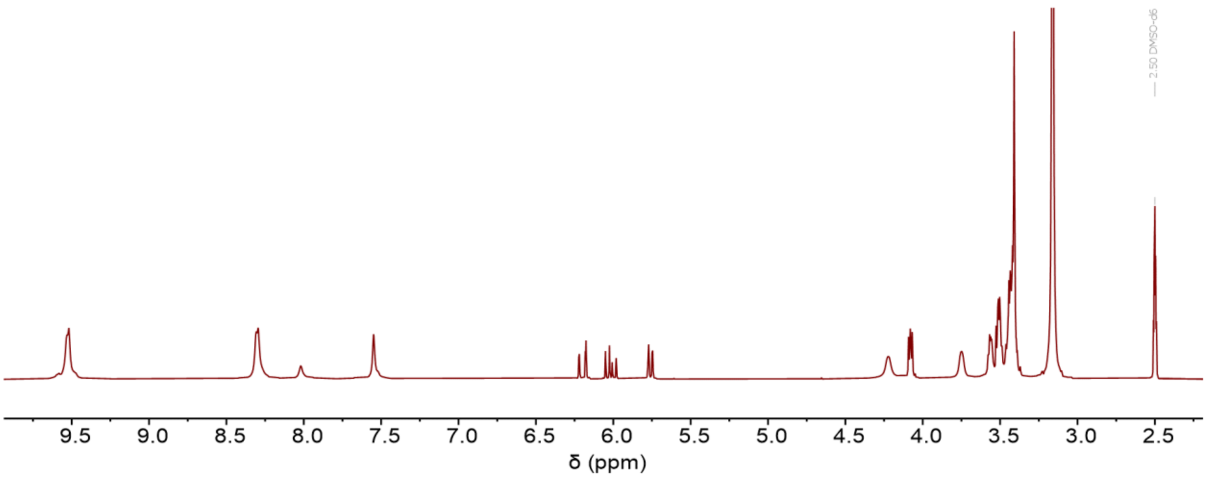

Figure S31. ^{1}H NMR spectrum (400 MHz, DMSO-d$_6$) of cage $[Pd_{12}(\mathbf{L2})_{24}](NO_3)_{24}$.

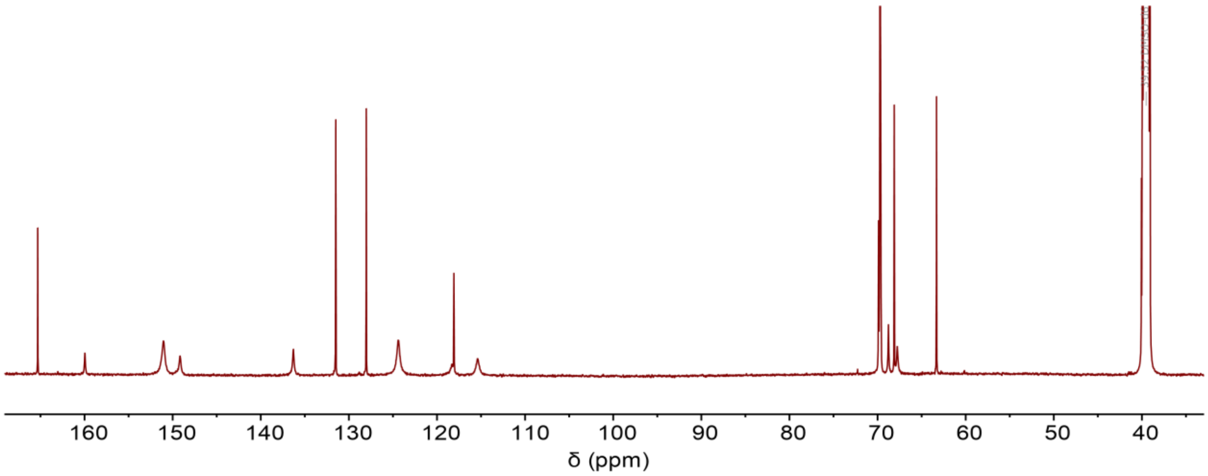

Figure S32. ^{13}C{^{1}H} NMR spectrum (151 MHz, DMSO-d$_6$) of cage $[Pd_{12}(\mathbf{L2})_{24}](NO_3)_{24}$.

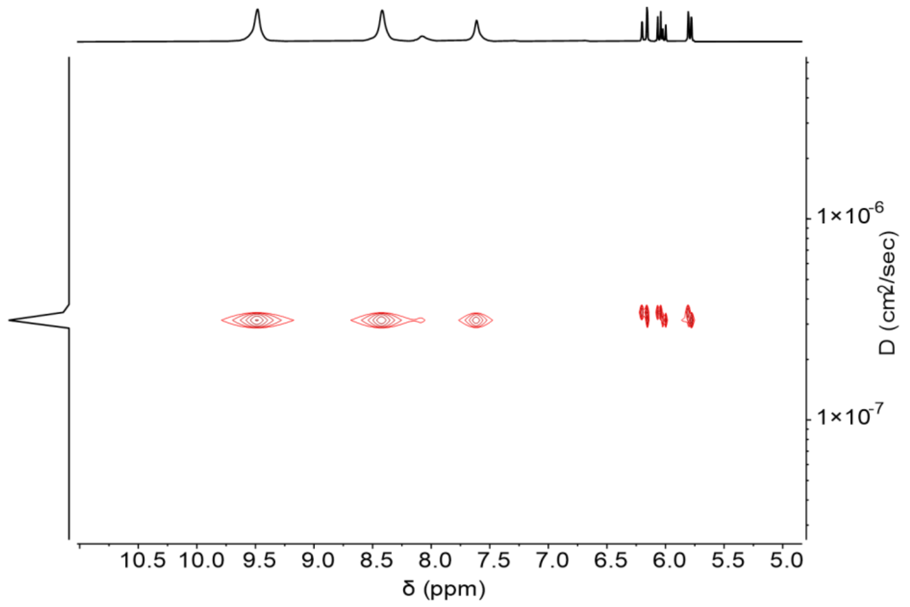

Figure S33. DOSY NMR spectrum of cage $[Pd_{12}(\mathbf{L2})_{24}](NO_3)_{24}$.

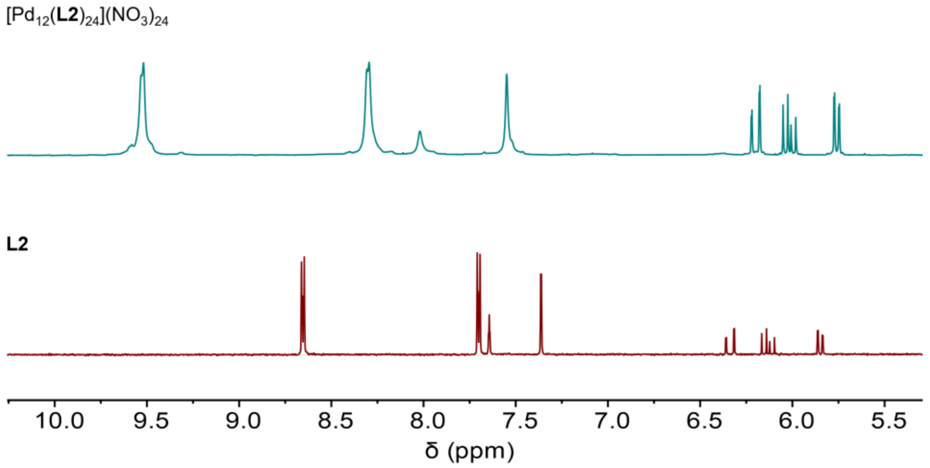

Figure S34. ^{1}H NMR spectrum (400 MHz, DMSO-d$_6$) of cage $[Pd_{12}(\mathbf{L2})_{24}](NO_3)_{24}$ and **L2**.

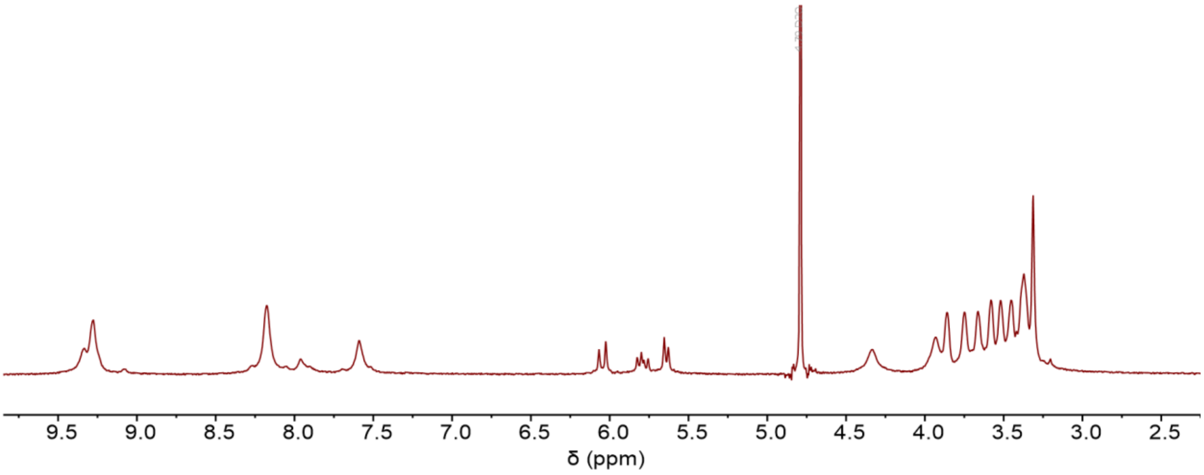

Figure S35. ^{1}H NMR spectrum (400 MHz, D_2O) of cage $[Pd_{12}(\mathbf{L2})_{24}](NO_3)_{24}$.

2.6 Synthesis and characterization of the hydrogels HG1–HG3

The cage-crosslinked hydrogels **HG1**–**HG3** were synthesized via photoinitiated radical copolymerization. Specifically, monomer DMA (19.83 mg, 200 μmol), initiator VA-86 (3.0 μmol, 1.5 mol%) and different amounts of cage $[Pd_2(\mathbf{L1})_4](NO_3)_4$ (1.25 mol%, 0.63 mol%, or 0.31 mol%,) were dissolved in D_2O (500 μL) in a 1.5 mL glass vial. The mixture was purged with N_2 for 30 min, then irradiated by a UV lamp for 1 h. After 12 h of incubating at RT, stable hydrogels were obtained. The hydrogels were characterized by ^1H NMR spectroscopy. The mechanical properties of the hydrogels were examined by rheometry. The swelling capability of the hydrogels was examined as described in section 1.2.

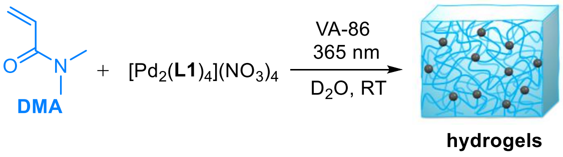

Scheme S7. Synthesis of the cage-crosslinked hydrogels **HG1**, **HG2**, and **HG3**.

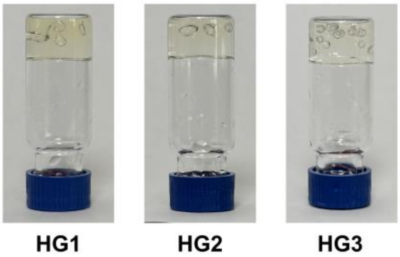

Figure S36. Pictures of the cage-crosslinked hydrogels **HG1**, **HG2**, and **HG3**.

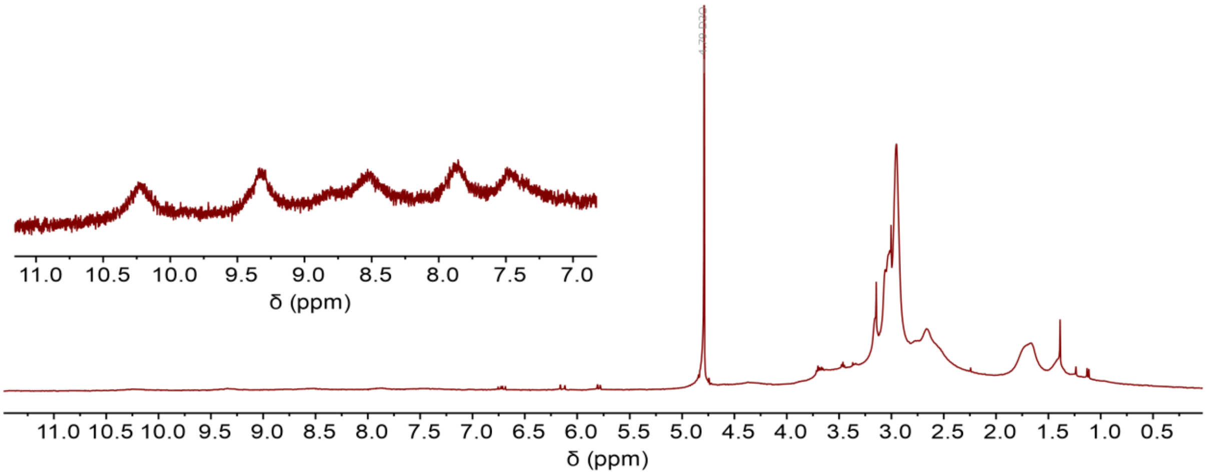

Figure S37. ^1H NMR spectrum (400 MHz, D_2O) of cage-crosslinked hydrogel **HG1**.

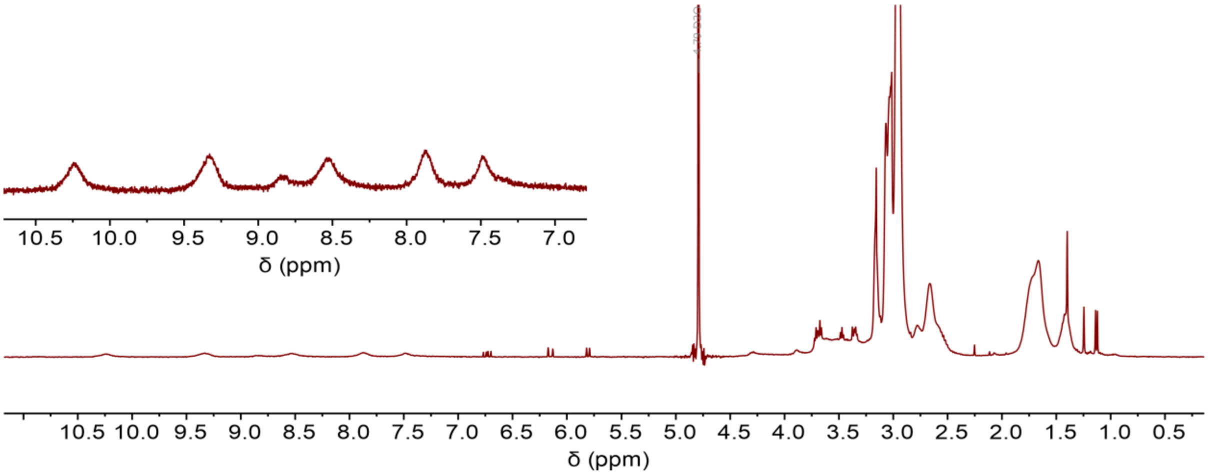

Figure S38. ^{1}H NMR spectrum (400 MHz, D_2O) of cage-crosslinked hydrogel **HG2**.

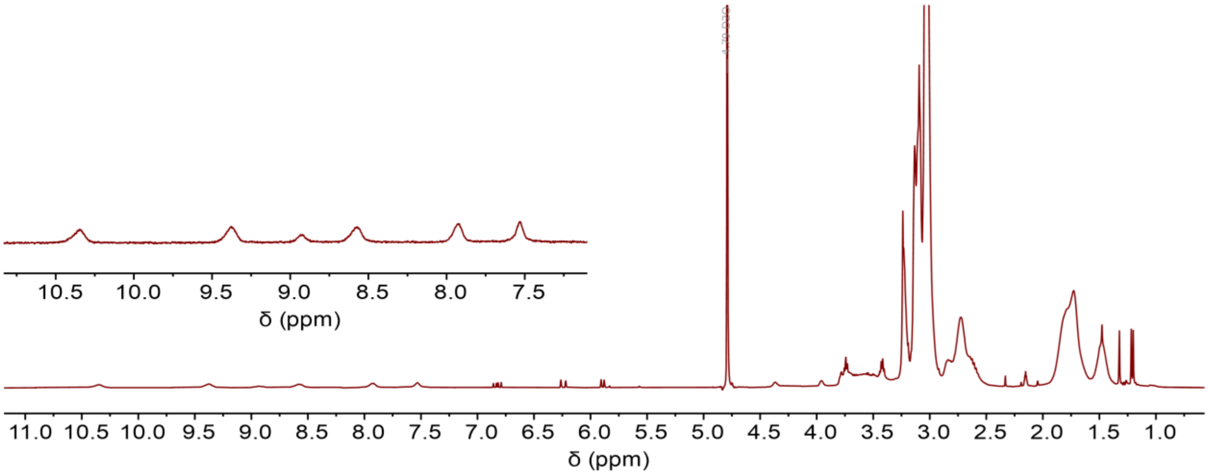

Figure S39. ^{1}H NMR spectrum (400 MHz, D_2O) of cage-crosslinked hydrogel **HG3**.

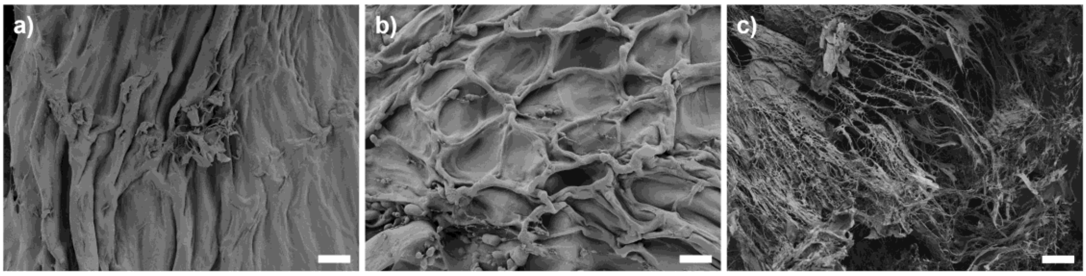

Figure S40. SEM images of **HG1** (**a**), **HG2** (**b**), and **HG3** (**c**), scale bar 50 µm.

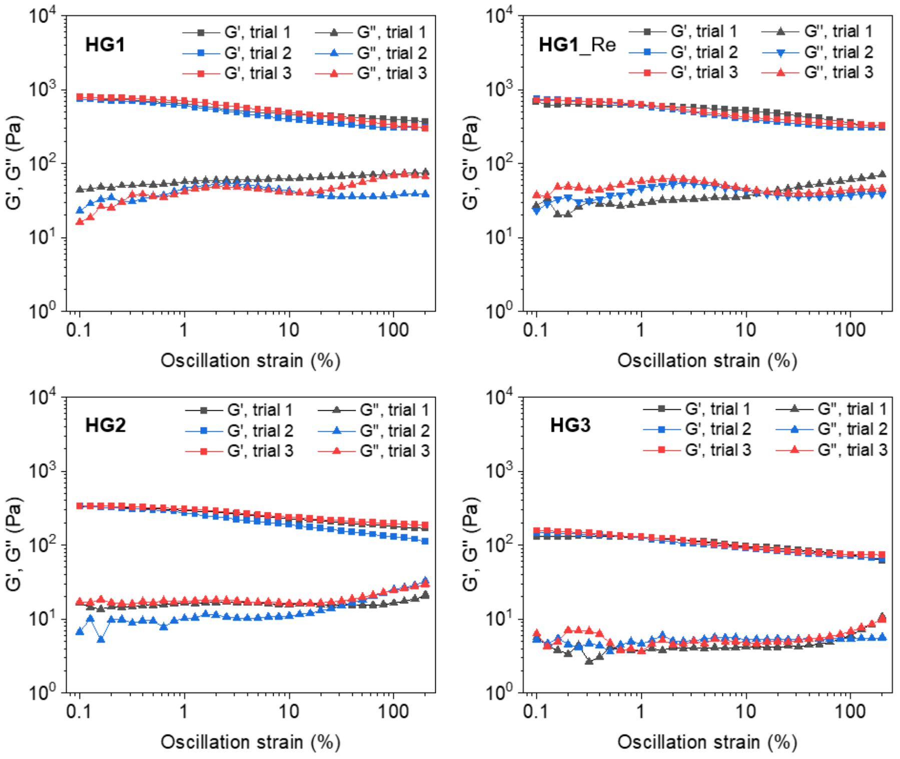

Figure S41. Amplitude sweeps in oscillatory rheology from 0.1% to 200% strain at frequency of 1 rad s^{-1} for **HG1**–**HG3** at 25 °C. The gel **HG1** was re-measured after standing at RT for one week (**HG1**_Re).

Table S1. Synthesis and characterization of **HG1**, **HG2**, and **HG3**.

	Monomer	Cage-crosslinker	Storage moduli (G')	Swelling capacities
**HG1**	DMA (200 µmol)	$[Pd_2(\mathbf{L1})_4](NO_3)_4$ (2.50 µmol)	742.8 Pa	42
**HG2**	DMA (200 µmol)	$[Pd_2(\mathbf{L1})_4](NO_3)_4$ (1.25 µmol)	331.9 Pa	35
**HG3**	DMA (200 µmol)	$[Pd_2(\mathbf{L1})_4](NO_3)_4$ (0.63 µmol)	129.7 Pa	31

2.7 Synthesis and characterization of hydrogels HG4 and HG5

For the synthesis of **HG4**, DMA (200 µmol) was used as monomer and cage [Pd_4(**L1**)$_8$] (BF_4)$_8$ (1.25 µmol, 0.63 mol%) was used as crosslinker. For the synthesis of **HG5**, DMA (200 µmol) was used as monomer and cage Pd_{12}(**L2**)$_{24}$](NO_3)$_{24}$ (0.42 µmol, 0.21 mol%) was used as crosslinker. The polymerization was carried out as described for **HG1**–**HG3**. The hydrogels were characterized by ^{1}H NMR spectroscopy. The mechanical properties of the hydrogels were examined by rheometry. The swelling capability of the hydrogels was examined as described in section 1.2.

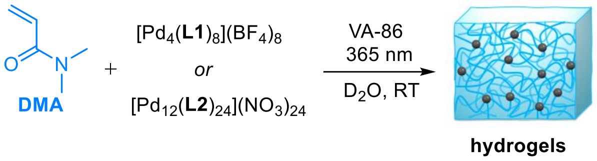

Scheme S8. Synthesis of the cage-crosslinked hydrogels **HG4** and **HG5**.

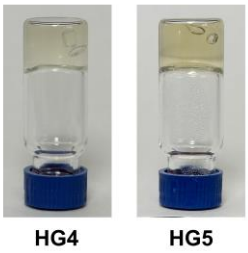

Figure S42. Pictures of cage-crosslinked hydrogels **HG4** and **HG5**.

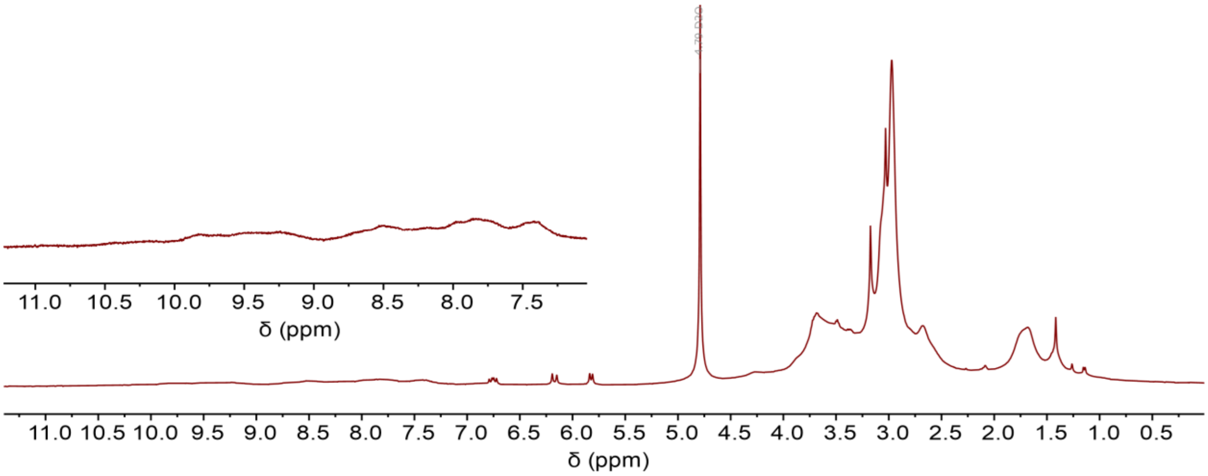

Figure S43. ^{1}H NMR spectrum (400 MHz, D_2O) of cage-crosslinked hydrogel **HG4**.

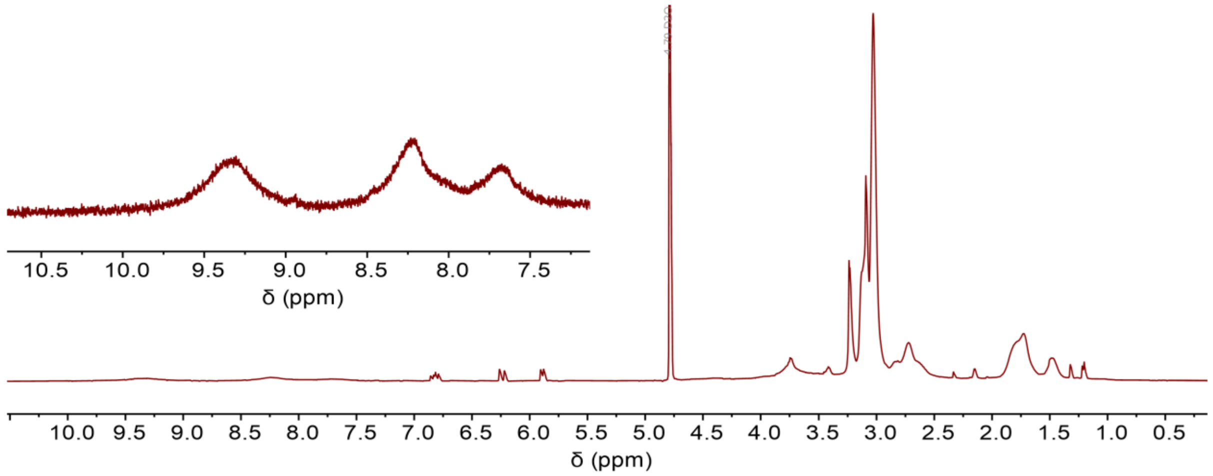

Figure S44. ^{1}H NMR spectrum (400 MHz, D_2O) of cage-crosslinked hydrogel **HG5**.

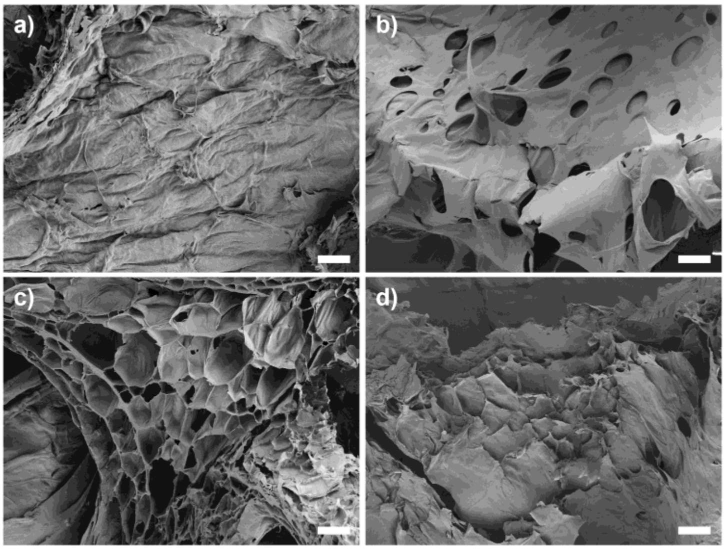

Figure S45. SEM images of **HG4** (**a**, **c**) and **HG5** (**b**, **d**), scale bar 50 μm.

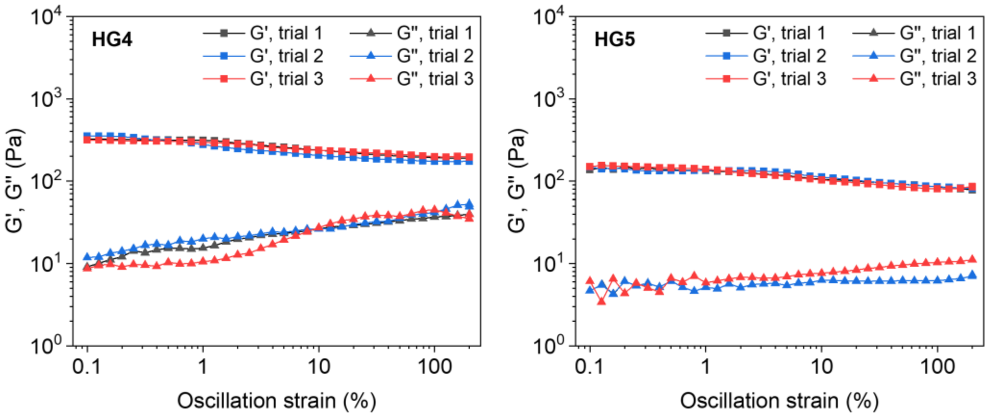

Figure S46. Amplitude sweeps in oscillatory rheology from 0.1% to 200% strain at frequency of 1 rad s^{-1} for **HG4** and **HG5** at 25 °C.

Table S2. Synthesis and characterization of **HG4** and **HG5**.

	Monomer	Cage-crosslinker	Storage moduli (G')	Swelling capacities
**HG4**	DMA (200 µmol)	$[Pd_4(\mathbf{L1})_8]$ $(BF_4)_8$ (1.25 µmol)	325.9 Pa	34
**HG5**	DMA (200 µmol)	$[Pd_{12}(\mathbf{L2})_{24}](NO_3)_{24}$ (0.42 µmol)	136.4 Pa	36

2.8 Synthesis and characterization of the hydrogels HG6 and HG7

For the synthesis of **HG6** and **HG7**, cage $Pd_2(\mathbf{L1})_4](NO_3)_4$ (2.50 µmol, 1.25 mol%) was used as crosslinker, while HEAA (200 µmol) and NIPAm (200 µmol) were used as monomers, respectively. The polymerization was carried out as described for **HG1**–**HG3**. The hydrogels were characterized by ^1H NMR spectroscopy. The mechanical properties of the hydrogels were examined by rheometry. The swelling capability of the hydrogels was examined as described in section 1.2.

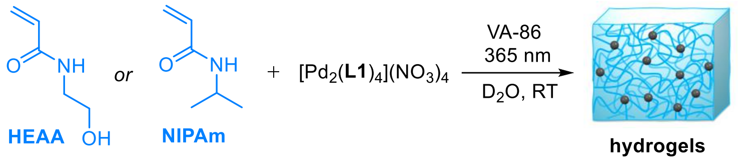

Scheme S9. Synthesis of the cage-crosslinked hydrogels **HG6** and **HG7**.

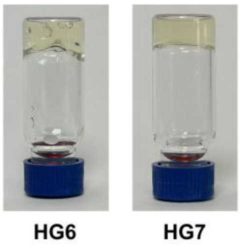

Figure S47. Pictures of cage-crosslinked hydrogels **HG6** and **HG7**.

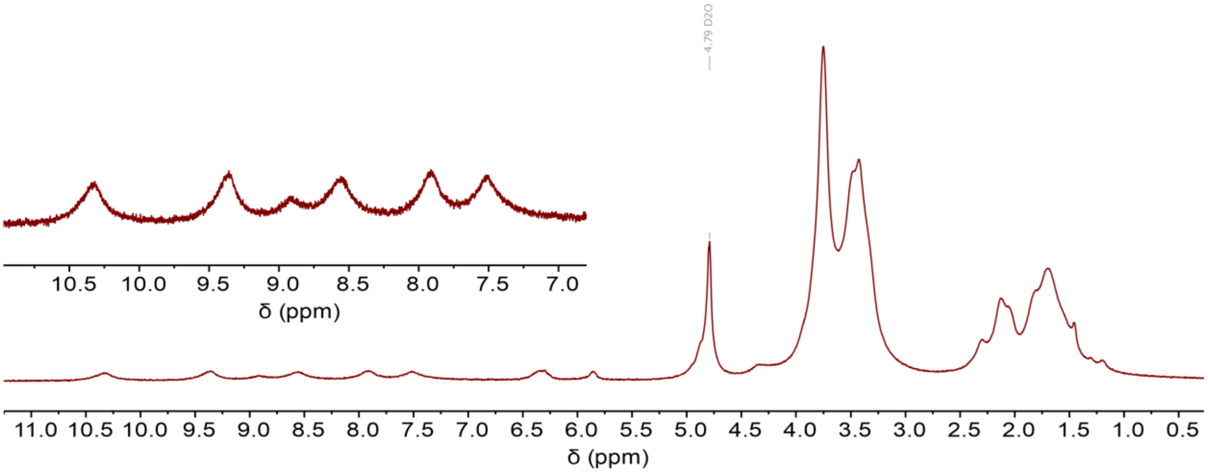

Figure S48. ^1H NMR spectrum (400 MHz, D_2O) of cage-crosslinked hydrogel **HG6**.

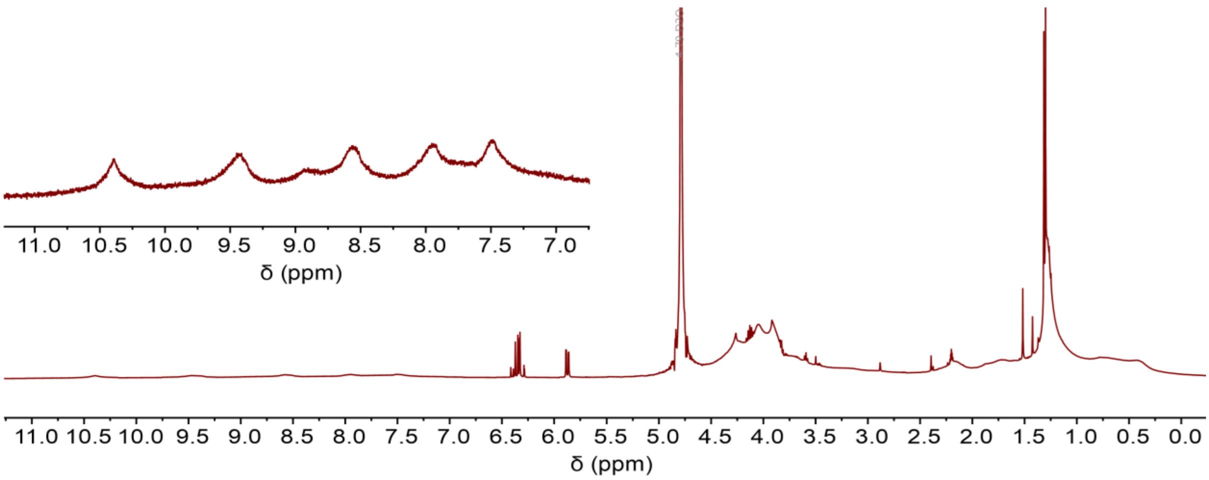

Figure S49. ^{1}H NMR spectrum (400 MHz, D_2O) of cage-crosslinked hydrogel **HG7**.

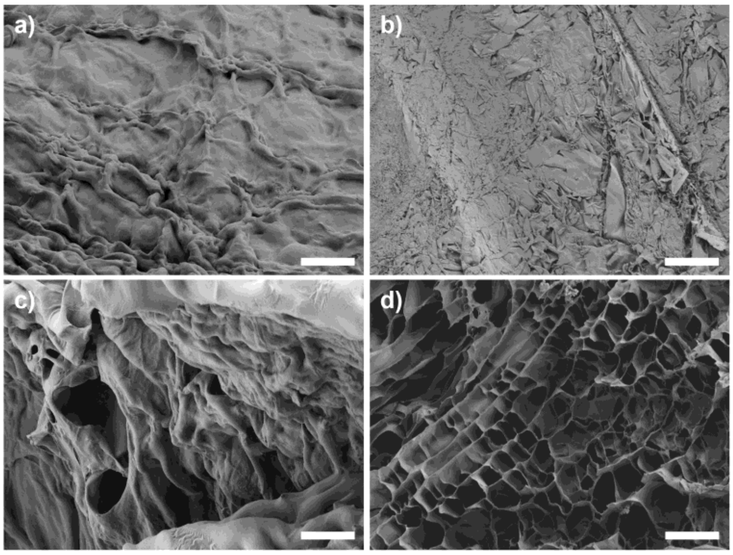

Figure S50. SEM images of **HG6** (**a**, **c**) and **HG7** (**b**, **d**), scale bar 40 µm.

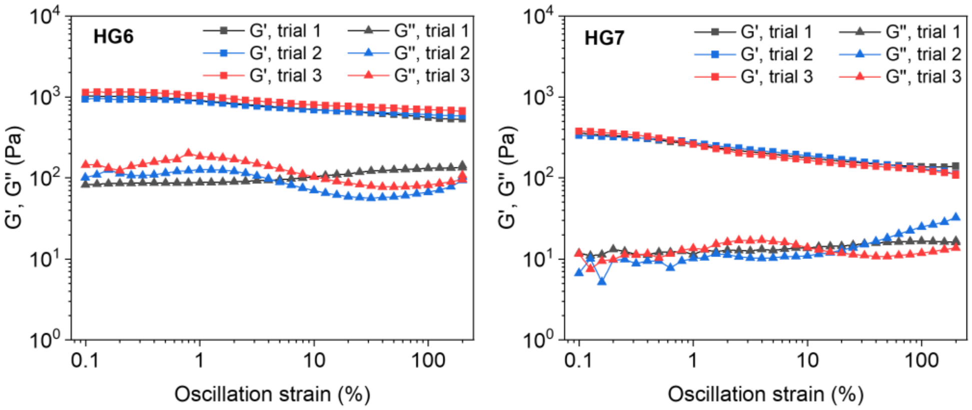

Figure S51. Amplitude sweeps in oscillatory rheology from 0.1% to 200% strain at frequency of 1 rad s^{-1} for **HG6** and **HG7** at 25 °C.

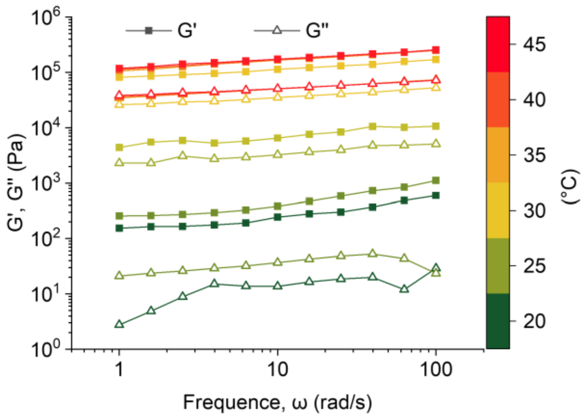

Figure S52. Frequency sweeps in oscillatory rheology from 1 to 100 rad s^{-1} at 1.0 % strain amplitude for **HG7** at different temperatures.

Table S3. Synthesis and characterization of **HG6** and **HG7**.

	Monomer	Cage-crosslinker	Storage moduli (G')	Swelling capacities
**HG6**	HEAA (200 μmol)	$[Pd_2(\mathbf{L1})_4](NO_3)_4$ (2.50 μmol)	1021.8 Pa	25
**HG7**	NIPAm (200 μmol)	$[Pd_2(\mathbf{L1})_4](NO_3)_4$ (2.50 μmol)	358.1 Pa	22

3 Chloride-triggered rearrangement of $[Pd_4(L1)_8]^{8+}$ into $[Pd_2(L1)_4Cl]^{3+}$

The chloride-triggered rearrangement of $[Pd_4(\mathbf{L1})_8](BF_4)_8$ was performed in an NMR tube and was tracked by ^1H NMR spectroscopy. Specifically, an NMR tube was filled with a solution of cage $[Pd_4(\mathbf{L1})_8](BF_4)_8$ in D_2O (500 μL, 2.5 mM), then 25 μL of a stock solution of NaCl (100 mM in D_2O, 2.0 eq.) was added and the mixture was equilibrated at RT for 30 min. ^1H NMR spectra were recorded before and after addition of NaCl.

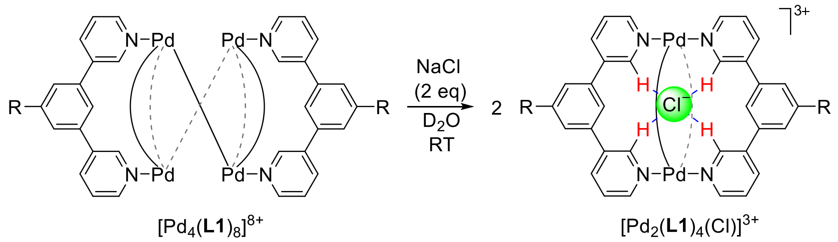

Scheme S10. Chloride-induced rearrangement of $[Pd_4(\mathbf{L1})_8]^{8+}$ into $[Pd_2(\mathbf{L1})_4Cl]^{3+}$.

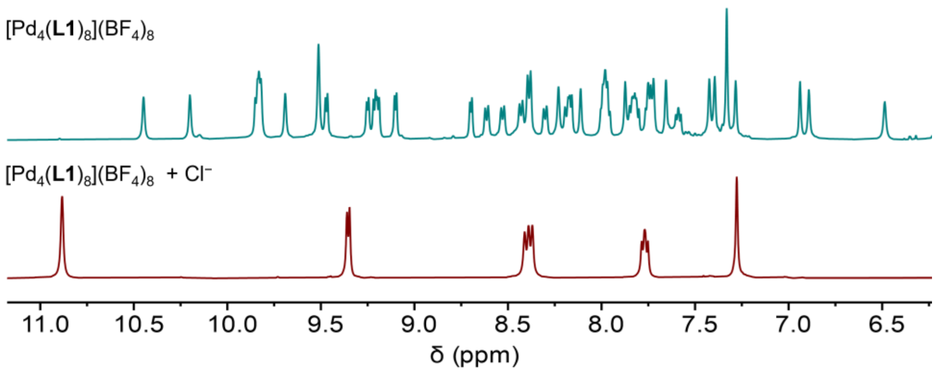

Figure S53. Aromatic region of the ^{1}H NMR spectrum (400 MHz, D_2O) of cage $[Pd_4(\mathbf{L1})_8](BF_4)_8$ before (top) and after addition of chloride (bottom).

4 Chloride-triggered topology changes of HG4

An aliquot (12.5 µL) of a NaCl stock solution (100 mM in D_2O, 2.0 eq.) was added to an NMR tube containing half of the hydrogel **HG4** synthesized as described in Section 2.7 (theoretically a content of 0.625 µmol of $[Pd_4(\mathbf{L1})_8]$ $(BF_4)_8$). The mixture was incubated at room temperature for 2 d. Subsequently, a ^{1}H NMR spectrum of the hydrogel was recorded, and the mechanical properties of the hydrogel were examined by rheometry.

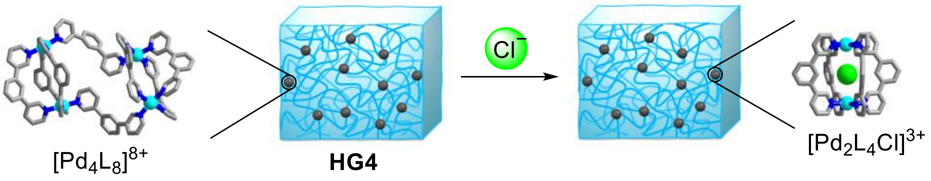

Scheme S11. Chloride-induced rearrangement of $[Pd_4(\mathbf{L1})_8]^{8+}$ into $[Pd_2(\mathbf{L1})_4Cl]^{3+}$in **HG4**.

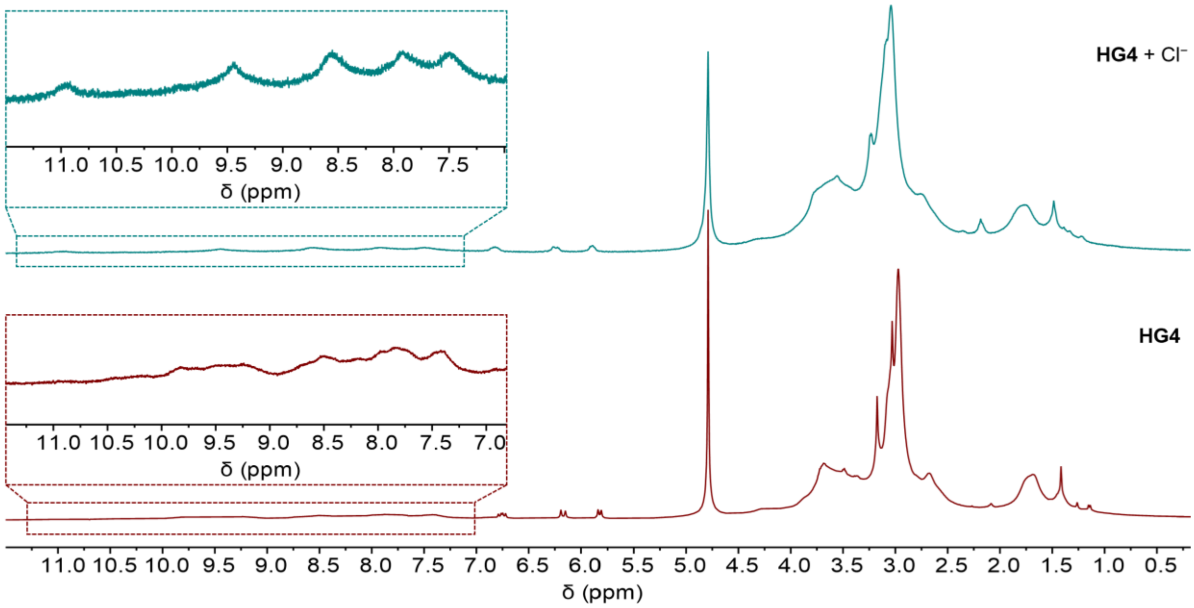

Figure S54. ^{1}H NMR spectrum (400 MHz, D_2O) of cage-crosslinked hydrogels **HG4** before (bottom) and after addition of chloride (top).

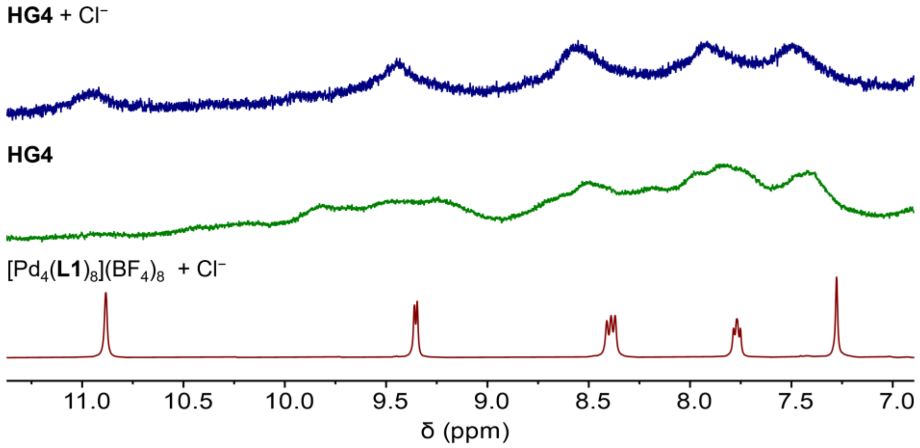

Figure S55. Aromatic region of the ^{1}H NMR spectrum (400 MHz, D_2O) of cage-crosslinked hydrogels **HG4** (middle), **HG4** after adding Cl^- (top), and cage $[Pd_4(\mathbf{L1})_8](BF_4)_8$ after adding Cl^- (bottom). The signal at ~ 11 ppm is indicative of the chloride-bound cage $[Pd_2(\mathbf{L1})_4Cl]^{3+}$.

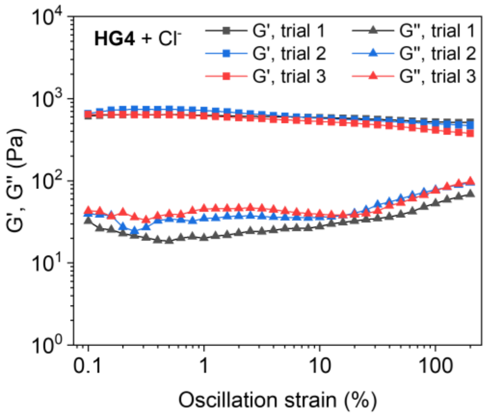

Figure S56. Amplitude sweeps in oscillatory rheology from 0.1% to 200% strain at frequency of 1 rad s^{-1} for **HG4** after treatment with chloride at 25 °C.

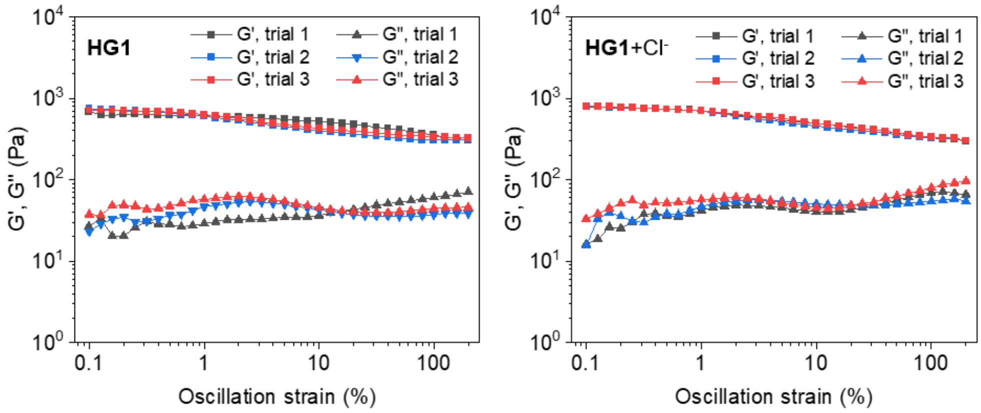

Figure S57. Amplitude sweeps in oscillatory rheology from 0.1% to 200% strain at frequency of 1 rad s^{-1} for **HG1** before and after incubation with NaCl (1 eq.) for 2 days at 25 °C.

5 Modeling detail

The starting geometry of $[Pd_4(\mathbf{L1*})_8]^{8+}$ was constructed using software Avogadro 1.2.0,[3] with **L1*** being the non-pegylated analogue of ligand **L1**. The cage was then optimized at the GFN2-xTB[4] level using software xTB[4] in implicit acetonitrile treated with the analytical linearized Poisson-Boltzmann solvent model (ALPB).[5]

Table S4. Coordinates of the xTB-optimized structure of $[Pd_4(\mathbf{L1*})_8]^{8+}$.

244
energy: -385.836591022043 gnorm: 0.000198069062 xtb: 6.6.1 (8d0f1dd)

Pd	5.44928992918874	-3.91758957675503	20.54286474274040
N	7.06437217722883	-3.73427479119156	19.37857952840802
N	7.94692231368368	3.63965389760521	21.12125368370700
C	8.48957051564496	-2.25217944116620	18.15345537787119
C	8.09320273495480	2.60317845332153	20.29369282463562
C	9.84670662034506	3.71833003527273	19.12797957172804
C	8.82813116760566	0.16404707945312	18.69723750488991
H	8.67010018369306	-0.05750694286336	19.74063602730463
C	9.37342796046793	1.73536196827801	16.96039877119301
C	7.64310671874557	-4.78687268114234	18.80279734036851
C	8.68548483678100	-4.63617071719890	17.90103879832332
C	9.09040010996313	1.47448880021570	18.30190632773355
C	8.71101946467786	4.72004986535032	20.97616586323489
C	9.68651087671932	4.78694314895412	19.99323870574316
C	8.83787490710742	-0.87450528945683	17.76876177433669
C	9.02218813515547	2.59429415093389	19.25466381671938
C	9.11089649591280	-3.36084452202924	17.56853342895292
C	9.12945938809911	-0.59352656314056	16.43383042772878
C	9.39753402636544	0.70443481659129	16.03879633903119
C	7.47404562620197	-2.50204170969620	19.07451561551297
H	6.95225066897394	-1.68410192028552	19.56733052400396
Pd	6.53055140604271	3.51116411937006	22.54166043675567
N	4.29246641399783	-3.27971314999638	19.03565277191291
N	5.12427880362321	3.83270727793226	21.15090095608710
N	5.07570343444842	3.25964881797485	23.89422439743139
N	3.83859431273723	-3.97942207453895	21.73877795426278
C	3.55068955151835	1.77226161020821	24.98399245693231
C	3.95163155479186	-4.08587511773308	18.03263947278278
C	2.55003634729939	-2.39251607959628	17.09095232981256
C	3.72371351976820	-2.99411687761878	22.63219521331645
H	4.50870889230395	-2.23891761372292	22.62366141912215
C	1.71230256802463	-3.93223050235930	23.49456048829676
C	3.09582830699598	-0.58029962972662	24.29681435285547
H	3.35579801990870	-0.31262281764114	23.28508875062021
C	2.25410878415609	-2.18238584803273	25.88167845555138
C	1.00393484614171	0.05344753925259	17.99253685223465
C	4.37640497713100	2.88719438150740	20.58806788367766
H	4.54617369481541	1.87417597060719	20.93922698083926
C	3.79015043772236	-2.04861374954804	19.09568258459544
C	4.42503163693462	4.30943982943635	24.39220351064944
C	4.95654687207940	5.10849195883428	20.80175666229966
C	3.31790249385951	4.15459128816773	25.21266676752136
C	2.58244439078717	2.09570206886327	19.04648253700582
C	3.24369607754115	4.50424721473930	19.25126969567285
C	3.40740444243538	3.16714785877296	19.62472089648053
C	2.66574195290346	-1.87459162350569	24.58498365929152
C	2.92236210456572	-4.94333116962408	21.69390669589928
C	2.33848234791818	-0.19813511060444	18.30752167142821
C	1.83336619212255	-4.94140616738329	22.55392278088291
C	3.10852943891968	0.39989656931844	25.28605346514717
C	2.68567022261650	-2.92875207206573	23.55729299295497
C	2.87324279805483	2.87760563841743	25.50882316645889

C	2.90054043195032	-1.54765692018393	18.14814979217936
C	1.24218763137790	2.32623971676175	18.73567593392884
C	3.12125094285755	0.83137028346079	18.82401593750111
H	4.17345178051036	0.66574666009383	18.99155070161751
C	2.69283285410325	0.07378869079059	26.57689098600479
C	2.27232279736156	-1.21179358126461	26.86737203788373
C	4.65994978318847	2.02256802324154	24.17782327271151
C	0.46610568092926	1.31038494763575	18.20637981955304
C	4.02276226515551	5.48146003724109	19.84880575285109
C	3.08325409472909	-3.67005933761552	17.03477920450368
H	9.54906050886675	2.74509728915510	16.62349897813004
H	8.54757342477529	5.54691734766600	21.66055246273180
H	9.12090471794370	-1.37853936848013	15.69368216129414
H	9.61632054365228	0.91662413082187	15.00332415005963
H	4.38053505466632	-5.08369518741977	18.02478215163678
H	1.93601964872595	-3.18345533755181	26.12602923948914
H	0.37603191196893	-0.73301631355479	17.60333657055746
H	4.78719623332705	5.29958432856065	24.12954068285106
H	0.79468938762099	3.28991967056659	18.92299429143150
H	2.71429756717542	0.81858957209401	27.35704929596589
H	1.95231763263350	-1.45645286742108	27.86827599408855
H	-0.56888163380628	1.49827526248143	17.96443452132596
H	2.82486762876824	-4.33603644230474	16.22717680009286
H	1.87912896091477	-2.05118388267717	16.31823004451709
H	4.08952513429686	-1.44644267259050	19.95031129329725
H	5.58423247298171	5.85117483224800	21.29062603200062
H	2.52604235166249	4.77547103617523	18.49289621875768
H	9.92110313316745	-3.22465685307050	16.86941038586420
H	7.26618610555623	-5.76989001621612	19.06870804469558
H	7.42222636664036	1.76236749816953	20.46276158372289
H	10.61030377736606	3.75300780768831	18.36686552688678
H	5.25737691359584	1.21075870501155	23.76394669704856
H	1.99826038748194	2.73994787811622	26.12506813825760
H	0.86587998124500	-3.91608035013547	24.16334928227386
H	3.05933370958677	-5.72918777380579	20.95828989856678
H	1.09395953116256	-5.72333460629659	22.48832881923632
H	2.80936629464441	5.02075833098078	25.60453185482076
H	10.30661228485406	5.66439284986873	19.90444701444096
H	9.15059086276907	-5.50168915736454	17.45718622789946
H	3.90775971404954	6.51814945972427	19.57584701313651
H	17.63925574275745	-5.36796394298702	27.66228320435668
H	15.92507922844414	-3.67868253250582	28.30750781670153
H	12.54957869307414	-0.83502199078410	29.32952735851863
H	15.63434793834459	-2.43644947265333	33.12540453553671
H	14.70508331789025	-2.20889713677283	30.82327671453475
C	16.61076040655647	-5.66149995914770	27.79795071768759
C	15.65877565956994	-4.72071351019448	28.16233088276342
H	10.72062924649877	0.59886663362319	30.21745046326880
C	11.65399031076803	-1.27259546403246	29.75955664989144
C	14.50036835013659	-3.08881564283221	31.42777024883043
C	15.01837375361031	-3.21611603476055	32.70686835873976
C	10.64029139239573	-0.47499800248353	30.26668308626068
C	16.22422769874047	-6.97825111178789	27.61449701606902
H	16.95830345044193	-7.72368330500600	27.35111704170960
Pd	13.01738258503678	-3.77575842097331	29.04074797462599
N	14.38665118082488	-5.06097464797774	28.34901313276449
N	13.73252324854044	-4.03611031104024	30.89151223212684
N	11.56805579879792	-2.60237993466464	29.78021189942487
C	14.73102971606584	-4.35563706775371	33.44066112830099
C	9.53634013326730	-1.07593901737766	30.84616330824024
H	15.10897586807501	-4.46260179464505	34.44538142075167
H	8.73365667147488	-0.47118206919662	31.23779747182983
C	14.88289369886794	-7.33675649245530	27.79182043907959
C	14.00276991030514	-6.32737251847644	28.17397472890251
H	15.71891734392754	-9.01446511019745	25.86684088355230
C	13.45708256744651	-5.13759050695683	31.58419885361101
C	10.50907501223585	-3.19335176707222	30.33770373860952

C	13.93324526552335	-5.35493925963421	32.87585173112312
C	9.46537942642453	-2.47215193556010	30.91578383052589
H	12.94458724686100	-6.53776129943385	28.32644886555089
C	14.96803889707154	-9.45203614144741	26.50596957075223
C	14.41657630446348	-8.71341493164527	27.55330054777811
H	15.53019695832130	-6.76530912598057	34.55148707315374
H	12.84908691772720	-5.88375897926158	31.07991112091701
H	10.51338858196350	-4.28175875333482	30.33802834843213
H	8.25551637567274	-1.62338935781651	33.14696061876310
C	13.61090742634219	-6.59882839652526	33.58991183312163
C	14.55224871846181	-7.20271822525703	34.42259460907191
C	8.36519493771222	-3.14530620096890	31.62572933052122
C	7.85334055784796	-2.55577991750595	32.78295435266604
C	14.54154863377557	-10.74508600201432	26.26253974953698
H	14.97370360549456	-11.30949413099789	25.45056117005337
C	13.42380016732805	-9.29137181978279	28.34337109605186
H	13.03016541424380	-8.75337543574632	29.19131326933889
C	12.36760545122995	-7.20289341394380	33.42074722887963
C	7.85762296624849	-4.37343018253336	31.20545382561103
C	14.24801408038119	-8.38633135815737	35.07166779634906
H	11.60729540039173	-6.70711494666961	32.83870787879866
H	8.20670731121685	-4.81282647948520	30.28438463630507
C	6.85053817353165	-3.18083497227302	33.50080971456420
H	14.98128005398602	-8.84805784686613	35.71537630722709
C	13.56897708913018	-11.32182889645485	27.05922892632856
C	13.00000391265770	-10.59694023082927	28.10603225013341
H	6.46383613281878	-2.72128369072295	34.39756492173174
C	12.06245466844968	-8.39877255693696	34.06377795611363
C	13.01387489467052	-8.98701565172827	34.89617571836929
C	6.85457404285125	-5.01003963976827	31.93310156956757
H	13.23976099650830	-12.32878767716089	26.85437611328520
C	6.35165866475649	-4.40124420822808	33.08370600872733
H	10.64077959971200	-9.50540808374811	29.00519405365561
H	10.66161100152752	-8.57134795379029	31.73390134473877
C	11.95750710464002	-11.22807868540670	28.93440310306787
H	8.27054736852874	-7.12791566366749	30.92967754241175
C	10.80591490021501	-10.54055433391714	29.30774784053092
H	12.80423167575771	-9.92309485583424	35.39040077237166
C	10.76426862796824	-9.05288854503292	33.84167707226606
C	10.17933076231480	-9.05760080768141	32.57615714658977
C	6.35235456452651	-6.34512082007369	31.56690735470590
C	7.21689112671953	-7.33707770186564	31.10897542079298
H	5.59399251027766	-4.89221041683776	33.67435497409021
C	12.07205833411596	-12.55964063082912	29.34922539619657
H	12.95329313868528	-13.13211683460341	29.10419443907213
N	9.82865517613146	-11.10859087238802	30.02042024705133
Pd	8.26036487989299	-9.93601858908489	30.48439398549065
N	9.02861643843392	-9.67437904178180	32.31834142944017
C	10.08510518867966	-9.71678855611116	34.86663636168785
N	6.82182262235438	-8.59663906355762	30.89885255013893
C	5.00994392003836	-6.69664319187313	31.74788599747938
H	10.48419592913392	-9.72607870106916	35.86890928839696
H	4.29083357985871	-5.95918531041803	32.06860579663243
C	11.05518267936437	-13.13458258562355	30.09163649180419
C	9.93759046073078	-12.37566926871537	30.40747664746003
C	5.54940888176186	-8.93406243989910	31.10296005198389
C	8.38764992186747	-10.31701423989677	33.29613698946760
C	4.60791938549039	-7.99875357656891	31.50707709518044
C	8.88523805624969	-10.35145637802685	34.58884918392040
H	11.12511433621794	-14.15849266723584	30.42179340224845
H	9.12019900207780	-12.79675474753259	30.98355761095492
H	3.58005932640172	-8.29330205410340	31.64527258871128
H	5.26942755365172	-9.96913900297256	30.93685156252052
H	7.45137790163387	-10.81159160164480	33.04371759215411
H	8.34566834679101	-10.86931545015331	35.36548308008766
H	11.28117801901594	-5.20080165561427	27.08137159696895
H	10.18093172403375	-4.57833112943031	24.92513304600218

C	11.46017063809498	-4.24077584604717	26.60531270888234
C	10.83233582570420	-3.88043938389128	25.42472926072504
N	12.31941058844026	-3.42106076731172	27.21496569901004
C	11.04603960148697	-2.61419878283644	24.90304885789395
H	10.55036060546947	-2.29014701377536	24.00171778907078
C	12.58800152661854	-2.22765734148379	26.68576002969989
C	11.93995597231110	-1.75799563191534	25.54592832213444
H	14.30389583259526	-0.57853389365872	24.94426250649826
H	13.32886134397270	-1.60974475327059	27.19091779544989
C	12.16929856241292	-0.37438377743694	25.09321564777633
C	13.46059716614497	0.08923320465925	24.85845648142739
C	11.08540730724149	0.48910387235867	24.96155588701343
H	10.08842605135645	0.13811030898819	25.17769118879337
C	13.66325484287913	1.40676448049838	24.48626275097928
H	14.66152426021059	1.76361937120186	24.28394570776666
C	11.29499469079116	1.82683225098022	24.64394665893924
C	12.59205410661693	2.27780174624551	24.39789610943146
C	8.98140690875092	2.54300972399986	23.95915941337571
H	12.76453039960554	3.30752906017647	24.12444104474235
C	10.18339102555327	2.79362332815147	24.61931640684216
N	7.99989354668679	3.44482607135295	23.90297095123406
C	10.32981066545125	4.02634888504959	25.26149731768131
H	11.24022020345962	4.25853420164498	25.79208269039568
C	8.14378871022512	4.62218411329221	24.51969929172642
C	9.29312460022029	4.94297309839096	25.22287177533083
H	7.31817184903698	5.33110327113996	24.45605589472439
H	9.37975118844298	5.89704752998983	25.71760296432034
H	8.17848851270488	-8.98630708412485	27.52868347274730
H	8.01321562642646	-5.86628290387504	25.35843051647992
N	7.40544068933515	-10.44719483355845	28.75326119322299
C	7.56819530209910	-9.88334417851256	27.55883863652152
H	8.00103885193055	-10.67561191979347	21.90706064730990
H	7.84808686353094	-3.42175686360378	24.91906129982461
C	7.74371717178258	-10.04520210016720	22.74426110087757
H	7.64648713047146	-11.67798233958745	24.11660238390008
C	7.53890362579567	-10.61178346021851	23.98959893296719
C	7.59965254736181	-5.49977889282573	24.43244519843723
C	7.61573387292074	-8.67933644906356	22.56969351851874
H	7.79984280673092	-8.25572592715532	21.59534453120196
C	7.51481154236448	-4.13847287266912	24.18545943234218
C	7.22332156443131	-9.80394819740150	25.08155142140404
C	6.67537130258420	-11.56047991683993	28.87076085590720
C	7.00719861898654	-10.42048320064605	26.40051289644297
H	6.57000661143046	-11.99345954873722	29.86747095118327
C	7.28966348305097	-7.85921954215256	23.65032497741987
C	7.10678525363563	-8.42890430630259	24.90840541603834
C	7.16338552069802	-6.40674645299516	23.46338994941031
H	6.80960774581928	-7.81515939439773	25.74445313545887
C	6.07338027336947	-12.15566653684998	27.77503125607956
C	6.98790565844280	-3.70594073472772	22.98139526556644
C	6.24434640529919	-11.58361269107093	26.52511932034087
H	6.93289109846337	-2.65177252187253	22.71753774371191
C	6.60266146230633	-5.88204130147967	22.29732729553458
H	5.48481197701264	-13.05081392849409	27.89674582138784
N	6.52340445811855	-4.57206894979996	22.07554994120604
H	5.77938628752239	-12.02698604387408	25.65817636675621
H	6.18890347058410	-6.53824849232611	21.53341574472195
H	8.80796343095279	1.60584821882974	23.43972122471354

6 References

1. R.-J. Li, C. Pezzato, C. Berton and K. Severin, *Chem. Sci.*, 2021, **12**, 4981–4984.
2. S. Ursuegui, J. P. Schneider, C. Imbs, F. Lauvoisard, M. Dudek, M. Mosser and A. Wagner, *Org. Biomol. Chem.*, 2018, **16**, 8579–8584.
3. M. D. Hanwell, D. E. Curtis, D. C. Lonie, T. Vandermeersch, E. Zurek and G. R. Hutchison, *J. Cheminform.* 2012, **4**, 17.
4. C. Bannwarth, S. Ehlert and S. Grimme, *J. Chem. Theory Comput*., 2019, **15**, 1652–1671.
5. S. Ehlert, M. Stahn, S. Spicher and S. Grimme, *J. Chem. Theory Comput*., 2021, **17**, 4250–4261.
